# Emotion Regulation in the Association Between Posttraumatic Stress Disorder and Substance Use: A Systematic Review With Narrative Synthesis

**DOI:** 10.1177/15248380241306362

**Published:** 2024-12-30

**Authors:** Alice May Bowen, Robert Calder, Joanne Neale, Tim Meynen, Gail Gilchrist

**Affiliations:** 1 National Addiction Centre, King’s College London, London, UK; 2 Society for the Study of Addiction, London, UK; 3 South London and Maudsley NHS Foundation Trust, London, UK

**Keywords:** trauma, PTSD, alcohol and drugs

## Abstract

Posttraumatic stress disorder (PTSD) and substance use commonly co-occur and represent a unique clinical challenge. Current interventions show modest effect sizes and high rates of dropout highlighting the need to better understand the mechanisms underlying the PTSD-substance use association. Evidence suggests emotion regulation may be an important factor underlying this association. This systematic review aims to examine the role of emotion regulation in the association between PTSD and substance use and to provide an understanding of differences in emotion regulation based on gender, trauma type, and social factors. Systematic searches of Medline, Embase, PsycInfo, ASSIA, CINAHL, and Web of Science identified 33 studies that met the review inclusion criteria. While findings were mixed, the results largely suggest difficulties regulating negative and positive emotions are important in the PTSD-substance use association. Emotion regulation was elevated in individuals with PTSD-substance use disorder (SUD) compared to SUD only and among individuals with more severe PTSD and substance use symptoms. A small number of studies highlighted the role of emotion regulation difficulties over time and in relation to treatment outcomes. Preliminary findings suggested there may be differences in emotion regulation in PTSD-substance use based on gender, trauma type, and social factors, though this requires further examination. Limitations of the included studies include small sample sizes, cross-sectional designs, and a predominant focus on alcohol use. The findings largely support self-medication and negative reinforcement models of substance use and highlight the possible utility of integrated interventions focusing on emotion regulation for PTSD-substance use. Recommendations for further research are discussed.

## Background

Posttraumatic stress disorder (PTSD) has been consistently associated with increased substance use, and clinically PTSD and substance use disorder (SUD) commonly cooccur (Currie, 2021; Haller & Chassin, 2013; Roberts et al., 2022). Specifically, the lifetime prevalence of PTSD among SUD patients is estimated to be between 36% and 50%, compared to 5% to 11% in the general population (Brady et al., 2004; Greenberg et al., 2015). This comorbidity represents a clinical and public health concern, which is associated with increased symptom severity and elevated rates of relapse, overdose, and suicide, compared with either disorder alone (Roberts et al., 2016; Schäfer & Najavits, 2007). PTSD-substance use patients present with complex physical health and psychosocial needs and clinicians widely regard this comorbidity as difficult to treat (Drapkin et al., 2011; Norman et al., 2018; Roberts et al., 2020). A recent meta-analysis of psychological interventions for PTSD-substance use demonstrated small to modest effects on PTSD symptoms and substance use severity, with most findings falling short of clinical importance (Roberts et al., 2022). High rates of dropout and low uptake of treatment were observed across studies, and it was unclear whether sequential or concurrent treatment was most efficacious. Given the limitations of the existing treatment landscape, it is critical to further our understanding of this comorbidity. One promising approach is the identification of mechanisms underlying the PTSD-substance use association, which may represent useful targets for treatment, such as emotion regulation.

Emotion regulation broadly refers to the way individuals process and respond to their emotions (Sloan et al., 2017). Most definitions of emotion regulation focus on abilities or strategies and a recent meta-analysis highlighted the need to consider both, as they capture distinct elements of emotion regulation (Weiss, Kiefer, et al., 2021). Emotion regulation abilities refer to typical ways an individual understands and responds to their emotions (Tull & Aldao, 2015). This approach is exemplified in *Gratz & Roemer’s Broad Deficit Model*, in which emotion regulation is a multifaceted construct involving: an awareness and acceptance of emotions; the capacity to engage in goal-directed behavior and inhibit impulsivity when distressed; the use of appropriate strategies to modulate emotional responses; and a willingness to experience negative emotions as part of pursuing meaningful activities (Gratz & Roemer, 2004). In contrast, emotion regulation strategies as described in *Gross’s Process Model* involve ways individuals might reduce, strengthen, or maintain the experience of emotions based on their current needs (Gross, 2015). These strategies include cognitive reappraisal (reinterpreting a situation in a neutral or positive way to reduce its emotional impact) and expressive suppression (intentionally inhibiting or attempting to hide an emotional response) (Aldao et al., 2010; Gross, 1998).

Difficulties regulating emotions have been consistently associated with both PTSD and substance use, and therefore represent a potential mechanism underlying their cooccurrence. (Seligowski et al., 2015; Stellern et al., 2023; Weiss, Kiefer, et al., 2021). Theoretically, the susceptibility hypothesis suggests substance use may exacerbate vulnerability to PTSD, for example by reducing the capacity to manage emotions or process trauma (Chilcoat & Breslau, 1998; Kaysen et al., 2011). It has also been suggested that substance use may place individuals in high-risk situations increasing their risk of experiencing trauma and, consequently, PTSD (Brady et al., 2004). Alternatively, shared vulnerability models suggest common risk factors may underlie the development of both PTSD and substance use. For example, trauma may result in neurobiological changes which lead to emotion regulation difficulties and in turn confer risk for both substance use and PTSD (María-Ríos & Morrow, 2020). However, empirical evidence shows the greatest support for negative reinforcement models, which suggest substance use may be an attempt to “self-medicate” in response to distressing emotions, potentially leading to ongoing substance use and the maintenance of PTSD symptoms (Baker et al., 2004; Ehlers & Clark, 2000; Hawn et al., 2020; Khantzian, 1997). Indeed, a narrative review suggested emotion regulation difficulties may be linked to substance use to manage trauma-related distress among individuals with PTSD (Westphal et al., 2017).

Mounting evidence suggests emotion regulation may be relevant to the development, maintenance, and treatment of cooccurring PTSD and substance use. Emotion regulation difficulties have been shown to mediate the association between trauma and PTSD and between trauma and SUD, implicating emotion regulation in the etiology of both disorders (Kahl et al., 2020; Wolff et al., 2016). PTSD and substance use severity have also been positively associated with difficulties regulating emotions (Ehring & Quack, 2010; Garke et al., 2021). Furthermore, emotion regulation difficulties have been implicated in the causal pathway between PTSD and substance use and linked to adverse outcomes in people with PTSD-SUD (Dixon-Gordon et al., 2014; Tull et al., 2015). In treatment, emotion regulation skills have been shown to predict alcohol intake both during and following SUD interventions (Berking et al, 2011). Most studies have focused on negative emotions, but emerging evidence suggests difficulties regulating positive emotions may also be important. For example, difficulties regulating positive emotions have been shown to mediate the PTSD-substance use association (Weiss et al., 2019). Theoretically, positive emotions may be experienced as aversive due to the associated physiological arousal or, alternatively, substance use may be an attempt to enhance or maintain a positive emotional state (Goncharenko et al., 2019; Litz et al., 2000; Weiss et al., 2019).

Additionally, there may be gender differences in emotion regulation in the association between PTSD and substance use, with preliminary evidence suggesting some strategies may be important for men while others are more important for women (Bornovalova et al., 2009; Tripp & McDevitt-Murphy, 2015). Gender may influence emotion regulation due to biological differences, the impact of gender roles on cognitive development, and/or gender-based cultural expectations (Sanchis-Sanchis et al., 2020). Alongside gender, there may be differences in emotion regulation based on trauma type. Theoretically, early, chronic interpersonal trauma may interrupt emotional development, and this type of trauma is associated with increased emotion regulation difficulties, compared with single event or late onset trauma (Ehring & Quack, 2010). Increased emotion regulation difficulties have also been associated with interpersonal trauma among individuals with PTSD and with trauma severity in PTSD-SUD (Kahl et al., 2020; Raudales et al., 2019). Furthermore, emotions are elicited within the social context which may influence emotion regulation (English et al., 2017; Shuman, 2013). Social factors may induce or exacerbate psychological distress which is difficult to manage or provide resources, such as social support, which could promote effective emotion regulation (Marroquín, 2011; Silva et al., 2016). Indeed, difficulties with emotion regulation have been associated with weaker social relationships and reduced social support (English et al., 2013; Gross & John, 2003). The provision of safe accommodation has also been associated with the ability to utilize coping skills, including emotion regulation, learned in an intervention for PTSD-SUD (Fallot & Harris, 2005). As such, social factors including relationships, income, education, employment, and housing may influence emotion regulation in PTSD-substance use.

It is important to deepen our understanding of emotion regulation in co-occurring PTSD and substance use while considering other contributing factors. This could support the refinement of existing theory and potentially inform much-needed advancements in treatment. While a growing number of studies have examined emotion regulation in the PTSD-substance use association, there has been no systematic review to date. As such, it remains unclear how emotion regulation alongside specific abilities and strategies contribute to PTSD-substance use. Therefore, the aim of this review is to assess the role of emotion regulation in the association between PTSD and substance use. To address current gaps in the literature, studies considering (a) substance use across the spectrum, including SUD (b) positive and/or negative emotion regulation, and (c) emotion regulation strategies and/or abilities, are included. Moreover, given their potential impact on emotion regulation in the PTSD-substance use association, this review also explores the role of gender, trauma type, and social factors.

## Method

A systematic review with narrative synthesis—prospectively registered with the International Prospective Register of Systematic Reviews (8/11/22; PROSPERO 2022; CRD42022373777; tinyurl.com/3nk227cc)—was conducted according to the Preferred Reporting Items for Systematic Review and Meta-Analyses guidelines (Page et al., 2021).

### Search Strategy

Searches were performed in the following databases between 1980, when PTSD was introduced as a formal diagnosis (APA, 1980), and January 20, 2023: Medline, Embase, PsycInfo, ASSIA, CINAHL, and Web of Science. The searches were updated on February 14, 2024, to identify any additional studies and backward citation searching of included studies was conducted. Searches combined terms related to (a) emotion regulation; (b) PTSD; and (c) substance use. A full list of search terms is available in Supplemental Appendix A.

### Eligibility Criteria

The PICo (Population, phenomena of Interest, Context) model was used to define inclusion criteria, using the hierarchy assessment method of eligibility (Stern et al., 2020). Accordingly, studies were eligible for inclusion if (a) the study population was adults (aged 18 years+) who were using substances and experiencing PTSD symptoms OR who completed measures assessing substance use and PTSD symptoms; (b) the phenomena of interest in the study was general emotion regulation; and (c) emotion regulation was considered in the context of substance use *and* PTSD symptoms. Eligible study designs included longitudinal studies, cross-sectional studies, cohort studies, qualitative studies or trials published in peer-reviewed journals in English. Review and discussion articles, dissertations, conference abstracts, presentations, study protocols, case studies and book chapters were excluded. We chose not to include grey literature to ensure the scientific rigor of the included studies. Studies investigating tobacco-use only were excluded due to differing policy and treatment implications along with more robust existing evidence for an association between PTSD and other substances (e.g., alcohol), versus tobacco (Mengin et al., 2022). Studies of concepts related to, but distinct from, substance use (e.g., cravings) were also excluded.

### Screening and Data Extraction

Titles, abstracts, and full texts were assessed by AMB and RC against eligibility criteria, with disagreements resolved by a third reviewer, GG. References were managed in Endnote 20. Data were extracted by AMB and logged in custom extraction tables in Microsoft Excel, including study and population characteristics, information on trauma, PTSD and substance use, and study analysis and results. The second reviewer RC checked extraction for a random selection of 10 studies to assess accuracy.

### Methodological Quality

The methodological quality of included studies was assessed using The Mixed Methods Appraisal Tool (MMAT) (Hong et al., 2018). The MMAT includes criteria to assess the quality of empirical quantitative, qualitative, and mixed-methods studies. To aid discussion, each study was rated as either high (all criteria met), moderate (three or four out of five criteria met), or low quality (less than three criteria met). AMB appraised the quality of each study and a random selection of 10 studies were then quality assessed by a second reviewer (RC) to ensure accuracy and consistency. AMB and RC met to discuss and resolve any discrepancies. Studies were not excluded based on methodological quality.

### Narrative Synthesis Process

Data from the included studies were summarized based on narrative synthesis guidelines (Popay et al., 2006). This approach was selected due to the methodological heterogeneity of the included studies, which precluded meta-analysis. Tabulation was used to summarize key data from the included studies. Textual descriptions of studies were systematically produced and transformed using the principles of thematic analysis via coding in MAXQDA, according to the aims of the review. All studies were grouped by study design, population, substance, and the type of trauma studied to aid comparisons. Moderator variables and subgroup analyses were also examined, focusing on key differences between studies, gender, and trauma type. The following social factors were considered: relationships, income, education and employment, housing, and homelessness. Finally, the limitations of the synthesis were examined.

## Results

### Study Selection

**Figure 1.**
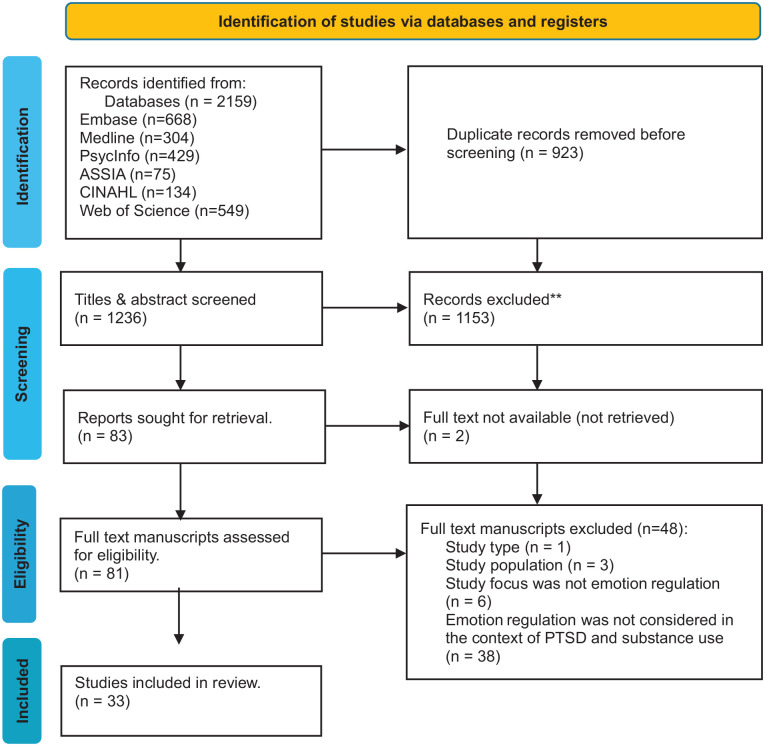
PRISMA diagram for this review. PRISMA = preferred reporting items for systematic reviews and meta-analyses.

As presented in Figure 1, following the removal of duplicates, 1,236 titles and abstracts were screened for eligibility and 81 full-text manuscripts were selected for review against the eligibility criteria. Thirty-three manuscripts were included in this review. The reasons for exclusion are presented in Figure 1. No additional manuscripts were identified through backward searching.

### Characteristics of Included Studies

A summary of the included studies is presented in Table 1, with further details in Supplemental Appendix B. All included studies were quantitative, most were conducted in the United States (*n* = 30, 90.9%), and most were cross-sectional (*n* = 28, 84.8%). Fifteen studies examined the indirect effects of emotion regulation in the association between PTSD and substance use. Five studies assessed variation in emotion regulation between groups based on PTSD, substance use, and/or trauma exposure. Four studies identified subgroups of participants based on PTSD, substance use, and/or emotion regulation difficulties, examining the relationship between these factors. Two studies tested emotion regulation as a moderator in the association between PTSD and substance use. One study examined emotion regulation as a predictor of alcohol dependence and PTSD, and one study examined PTSD and alcohol use as predictors of emotion regulation. Of the remaining five studies, three were prospective longitudinal cohort studies and considered relationships between emotion regulation, PTSD, and substance use over time. The final two studies were secondary analyses of Randomized Controlled Trials (RCTs) which examined emotion regulation in relation to PTSD and substance use outcomes. Most studies used self-report measures of emotion regulation, PTSD, substance use, and trauma. The measures used in each study are presented in Supplemental Appendix C.

**Table 1.** Summary of Key Findings From the Included Studies on Overall ER, Abilities and Strategies.

Study (*N*)	Authors (Year)	Sample (*N*), Sex/Gender^a^	Trauma, substance	Key Findings on Overall ER, Abilities, and Strategies
**Studies of indirect effects**				
1	Bornovalova et al. (2009)	Clinical (182), 72% male	Not reported, Alcohol and drugs	- For women, impulsivity when distressed partially explained the association between PTSD and substance use. For men, emotional awareness/clarity partially explained the association between PTSD and substance use
2	Goldstein (2017)	Trauma-exposed adults (260),100% female	Not reported, Alcohol	- ER had an indirect effect on the association between PTSD and alcohol dependence - An inability to pursue goals when distressed above other ER abilities had an indirect effect on the association between PTSD and alcohol dependence
3	Goncharenko et al. (2019)	Trauma-exposed adults (475), 43.2% male, 55.4% female, 0.8% female to male transgender, 0.1% male to female transgender	Various, Alcohol	- Positive, but not negative, ER had an indirect effect on the association between PTSD and alcohol use
4	Klanecky et al. (2016)	College (213), 63.4% men, 36.6% women	CASA, Alcohol	- The interaction between ER and dissociative tendencies was associated with increased alcohol use and PTSD symptoms
5	Klemanski et al. (2012)	Military (44), 100% male	Various, Alcohol	- ER did not have an indirect effect in association between PTSD and alcohol use
6	Leonard (2023)	Firefighters (685), 93.6% male, 5.7% female, 0.7% transgender	Various, Alcohol	- ER difficulties did not have an indirect effect on the association between PTSD and alcohol use
7	McGrew et al. (2023)	College (282), 77.5% female, 21.5% male	Various, Alcohol	- ER had an indirect effect on the association between PTSD symptom severity and alcohol use
8	Patel et al. (2023)	Healthcare workers (299), 91.6% female Public safety personnel (199), 39.7% female	Not reported, Alcohol and drugs	- ER difficulties had an indirect effect on the association between PTSD and drug, but not alcohol, use in healthcare workers and alcohol, but not drug, use in public safety personnel.
9	Paulus et al. (2019)	Trauma-exposed adults (238), 88.7% female	Various, Alcohol	- ER had an indirect effect on the association between PTSD and drinking severity and possible hazardous drinking, respectively.
10	Radomski (2016)	College (466), 53% female	Various, Alcohol	- ER had an indirect effect on the association between PTSD status and alcohol use. - Participants who met full or partial PTSD criteria reported more difficulty with impulsivity when distressed as a function of PTSD status and this in turn was associated with greater alcohol use.
11	Tripp and McDevitt-Murphy (2015)	Military veterans (139), 89% male	Military, Alcohol	- Overall ER and specific ER abilities did not have an indirect effect on the association between PTSD and alcohol use.
12	Weiss et al. (2020)	Military veterans (468), 70.50% male, 29.1% female, 0.4% female to male transgender	Not reported, Alcohol	- Positive emotional avoidance, but not positive emotional intensity, had an indirect effect on the association between PTSD symptoms and alcohol use.
13	Weiss, Goncharenko, et al. (2021)	Trauma-exposed adults (320), 50.3% men, 46.9% women, 2.8% different gender	Various, Alcohol	- Alcohol use to down-regulate negative and positive emotions both had an indirect effect on the association between PTSD and alcohol use, separately.
14	Weiss et al. (2019)	Trauma-exposed adults (463), 42.8% male, 55.7% female	Various, Alcohol and drugs	- Difficulties regulating positive emotions had an indirect effect on the association between PTSD symptom severity and alcohol, and drug use, respectively.
15	Wolitzky-Taylor et al. (2023)	Adults experiencing probable PTSD and hazardous drinking (513), 49.9% female	Various, Alcohol	- ER difficulties had an indirect effect on the association between PTSD and alcohol use.
**Moderation studies**				
16	Feingold and Zerach (2021)	Military veterans (189), 100% male	Military, Alcohol	- Cognitive reappraisal moderated the association between PTSD symptoms and AUD with a stronger association at high levels of cognitive reappraisal - Expressive suppression moderated the association between PTSD symptoms and alcohol use, with a stronger association at high levels of expressive suppression.
17	Mahoney (2022)	College with experience of attempted/completed rape (287), 100% women	Sexual abuse, Drugs	- For women with low ER difficulties combined with high coping self-efficacy, no association between PTSD and substance use
**Group comparison studies**				
18	Lebeaut et al. (2021)	Firefighters (657), 94% male, 6% female	Not reported, Alcohol	- PTSD-AUD participants, relative to trauma-exposed or AUD only participants, reported significantly more difficulties with ER but not than PTSD-only participants.
19	McDermott et al. (2009)	Clinical (58), 70.7% male	Various, Crack cocaine	- SUD-PTSD relative to SUD-only patients, reported significantly more difficulties with overall ER and all ER abilities, except awareness.
20	Wegen et al. (2017)	Clinical (243), 53.9% female, 46.1% male	Various, Alcohol and drugs	- PTSD only, relative to PTSD-SUD or SUD, participants reported significantly more difficulties with ER.
21	Weiss, Tull, Anestis, et al. (2013)	Clinical (205), 49.5% male	Not reported, Alcohol and drugs	- SUD-PTSD patients, relative to SUD-only patients, reported significantly more difficulties with overall ER and all ER abilities, except awareness.
22	Weiss, Tull, Lavender, et al. (2013)	Clinical (93), 76.3% male	Childhood and adolescent, SUD patients – substance not specified	- SUD-PTSD patients, relative to SUD-only patients, reported significantly more difficulties with overall ER, goal-directed behavior when distressed, impulsivity when distressed, limited access to ER strategies, and a lack of emotional clarity.
**Studies identifying subgroups**				
23	Christ et al. (2022)	Trauma-exposed adults (334), 63.5% male, 36.2% female	Various, Alcohol	- Cognitive reappraisal did not differ among profiles based on PTSD/AUD symptom severity. - Expressive suppression was elevated in more severe PTSD/AUD profiles
24	Lilly and London (2015)	Interpersonal trauma survivors (205), 100% female	Interpersonal, Alcohol	- Clinically distressed groups (clinical levels of PTSD, probable substance dependence) reported significantly more difficulties with overall ER and ER abilities except awareness, than other groups. - Non-acceptance and impulsivity were significant predictors of group membership.
25	Weiss et al. (2018)	Adults - experience of IPV (210), 100% women	IPV, Alcohol, and drugs	- The group characterized by difficulties regulating both positive and negative emotions (vs. low difficulty with ER) reported significantly more severe PTSD symptoms and alcohol use.
26	Witte et al. (2020)	College (946), 71.9% women, 26.2% men, 1.9% missing	Various, Alcohol	- Profiles characterized by PTSD symptoms and significant deficits in overall ER and all ER dimensions (except awareness) demonstrated elevated drinking frequency and quantity when compared with other profiles which were characterized by fewer ER difficulties.
**Other cross-sectional studies**				
27	Fairholme (2013)	Clinical (220), 47.7% female	Not reported, Alcohol	- ER was significantly related to alcohol dependence, PTSD, depression, and anxiety while controlling each of these and the interrelations among the variables. - No specific ER abilities (awareness excluded) were associated with alcohol dependence - Goal-directed behavior and impulsivity when distressed were significantly associated with PTSD symptom severity
28	Pebole et al. (2022)	Military veterans (74), 93.2% male, 5.5.% female, 1.4% missing	Not reported, Alcohol	- PTSD significantly predicted ER difficulties and along with alcohol use explained 37% of the variance in ER. - PTSD, but not alcohol use, significantly predicted all ER abilities, except awareness
**Prospective longitudinal studies**				
29	Aase et al. (2018)	Military veterans (71), 83.1% male	Military, Alcohol	- Cognitive reappraisal moderated the association between PTSD at baseline and alcohol use over one year. - No effects of expressive suppression
30	Tull et al. (2015)	Trauma-exposed adults (106),100% women	Various, Alcohol and drugs	- ER moderated the association between PTSD symptoms at baseline and substance use eight months later - Significant positive association between PTSD symptoms at baseline and alcohol use eight months later, for participants with low willingness to experience distress to pursue goals
31	Weiss, Brick, et al. (2022)	Adults - experience of IPV (145), 100% women	IPV, Alcohol, and drugs	- PTSD moderated the association between positive ER and drug, but not alcohol, use, over 30 days
	**Secondary analysis of RCTs**			
32	Hien et al. (2017)	Clinical (110), 36.36% female	Various, Alcohol and drugs	- ER moderated response to different psychological therapies for PTSD and substance use outcomes
33	Holzhauer and Gamble (2017)	Clinical (48), 100% women	Not reported, Alcohol	- Lower depression at the end of treatment, but not PTSD symptoms, mediated the association between improvements in ER during treatment and greater abstinence following treatment.

*Note.* AUD = alcohol use disorder; CASA = childhood and adolescent sexual abuse; ER = emotion regulation; IPV = intimate partner violence; SUD = substance use disorder; PTSD = posttraumatic stress disorder; RCTs = randomized controlled trials.

a Displayed as reported in included paper.

**Table 2.** Critical Findings.

• We reviewed 33 empirical studies of emotion regulation in the PTSD-substance use association published between 1980 and 2024, the majority were conducted in the US. • The studies used a variety of methodological approaches, largely providing evidence for the role of emotion regulation in the PTSD-substance use association. • Impulsivity when distressed and difficulties with goal-directed behavior were highlighted as particularly important emotion regulation abilities in the PTSD-substance use association. • A limited number of studies examined emotion regulation strategies, producing mixed findings. • Preliminary evidence suggests there may be gender differences in the specific emotion regulation abilities explaining the association between PTSD and substance use for men and women. • Preliminary evidence suggested different traumatic experiences may show different relationships to emotion regulation. • Preliminary evidence suggested social factors, and particularly relationships may be important in relation to emotion regulation in the PTSD-substance use association.

PTSD = posttraumatic stress disorder.

**Table 3.** Implications for Policy, Practice, and Research.

Focus	Implications
Policy	• Integrated treatment which focuses on both PTSD and substance use simultaneously should be prioritized by commissioners.
Practice	• Assessment of emotion regulation, including regulating positive emotions, at treatment entry may be useful. • Treatment focusing on emotion regulation could support improved outcomes in PTSD-substance use populations.
Research	• Quantitative research should consider using multimodal assessment of emotion regulation, PTSD, and substance use. • Prospective longitudinal research could delineate causation and temporality. • Further research is needed in relation to trauma type, gender, and social factors and should also consider other substances and polysubstance use. • Studies examining the context of emotion regulation are needed .• Qualitative studies could provide a more nuanced understanding of relationships between emotion regulation, trauma, PTSD, and substance use.

PTSD = posttraumatic stress disorder.

#### Sample and Demographic Characteristics

The 33 studies included 9,447 participants (50.5% women, 49% men, 0.2% transgender, 0.3% missing data) with sample sizes ranging from 44 (Klemanski et al., 2012) to 946 (Witte et al., 2020) and the mean age of participants ranging from 18.84 (Witte et al., 2020) to 45.43 years (McDermott et al., 2009). Twenty-four studies used mixed-gender samples while seven studies included samples of all-women and two of all-men. None of the included studies focused on gender minorities. Ethnicity was described in most studies (*n* = 30). Sample populations varied: eleven studies included community samples of trauma-exposed adults; eight included clinical samples, defined in this review as studies which recruited from community or inpatient substance use services; five included college samples; two included firefighters; and one included healthcare workers and public safety personnel. Most studies examined alcohol use (*N* = 21, 63.6%). The remaining studies examined alcohol and drugs (*n* = 9), drugs (*n* = 1) and crack cocaine (*n* = 1). One study included participants with SUD but did not specify the substance/s used by participants. Regarding trauma type, 16 studies included participants who had experienced various types of traumas, while nine did not report on the trauma experienced by participants. The remaining eight studies included participants who had specifically experienced: military trauma (*n* = 3), intimate partner violence (*n* = 2), childhood and adolescent trauma (*n* = 2), or sexual assault (*n* = 1). Supplemental Appendix B presents the full sample and demographic characteristics of the included studies. To note, 20 studies did not explain how gender was determined, nine studies said gender was self-reported, three studies said sex was self-reported and one study specifically said participants reported on sex at birth. Twenty-three studies used terms relating to natal sex (male/female) and gender (men/women) interchangeably. Throughout the synthesis, we use terms that refer to gender (e.g., men, women, transgender) in line with Sex and Gender Equity in Research guidelines (SAGER; Heidari et al., 2016) while recognizing it was not possible to determine if most of the included studies referred to sex or gender.

#### Quality Assessment

**Figure 2.**
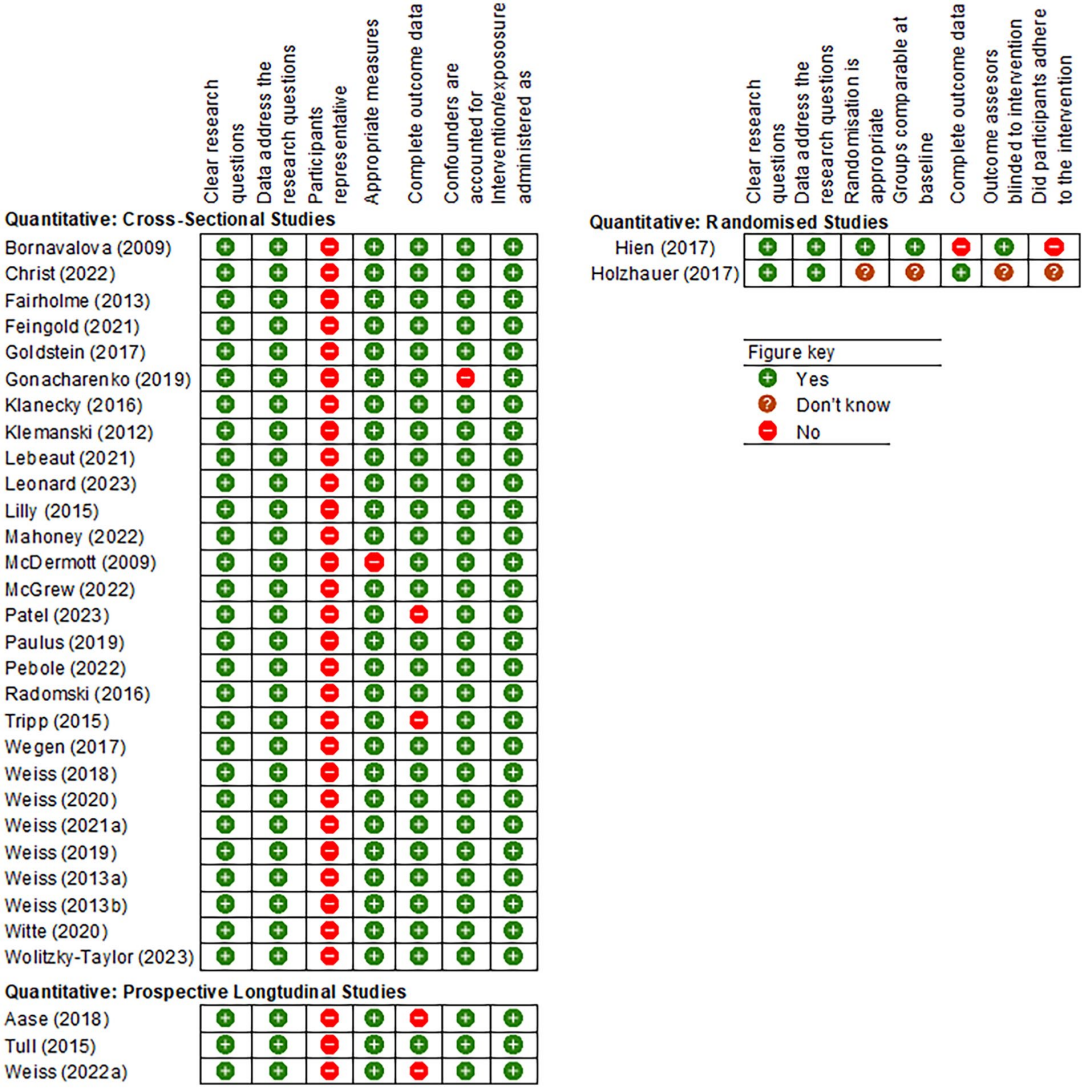
Final MMAT criteria and quality assessment of included studies by study design. MMAT = mixed methods appraisal tool.

Quality assessment found that the included studies were of moderate (*n* = 32) or low (*n* = 1) quality. The results of the quality assessment along with the criteria used to assess the studies are presented in Figure 2. Thirty-one studies were assessed according to the MMAT criteria for quantitative non-randomized studies. Most of these used appropriate and validated measures of PTSD, substance use, and emotion regulation (*n* = 30). However, there was a strong risk of bias regarding sample representativeness, precluding the generalisability of findings. None of the study samples were nationally representative of the target population. Instead, studies were often characterized by small, self-selecting samples meaning that analyses were likely underpowered to detect significant effects. In addition, four of the studies had high rates of attrition or missing data and one study did not account for possible confounding variables. The remaining two studies were assessed using the MMAT assessment criteria for quantitative randomized studies with the results presented in Figure 2.

### Narrative Synthesis

The following sections present a synthesis of the findings pertaining to emotion regulation abilities, emotion regulation strategies, gender, trauma type, and social factors. Key findings are presented in Table 1. Each study is numbered in Table 1, and corresponding numbers are used in text to reference studies. Additional findings relating to gender, trauma type, and social factors are presented in Supplemental Appendix D.

#### Emotion Regulation Abilities

Twenty-eight of the included studies examined emotion regulation abilities in the PTSD-substance use association. Most of these studies measured emotion regulation abilities using The Difficulties in Emotion Regulation Scale (DERS) which produces a total score and six subscale scores for specific emotion regulation abilities: emotional acceptance, difficulty engaging in goal-directed behavior when distressed, impulsivity when distressed, emotional awareness, access to emotion regulation strategies, and emotional clarity (Gratz & Roemer, 2004). The results are presented below according to study type.

#### Studies of Indirect Effects of Emotion Regulation Abilities

Fifteen studies examined the indirect effects of emotion regulation in the association between PTSD and substance use (Studies 1–15). Twelve of these studies focused on alcohol use (2–7, 9–13, and 15). Seven studies found that difficulties regulating negative emotions had an indirect effect on the association between PTSD and substance use (Studies 1, 2, 7–10, and 15). Additionally, in one study childhood and adolescent sexual abuse influenced alcohol use via PTSD and dissociative tendencies but not emotion regulation. However, the interaction between emotion regulation difficulties and dissociative tendencies was associated with increased alcohol use and PTSD symptomology (Study 4). Three of these studies considered specific emotion regulation abilities. One study of college students who had not experienced trauma or who had experienced trauma and met full or partial criteria for PTSD found that those in the PTSD group reported more difficulty with impulsivity when distressed as a function of PTSD status, and this, in turn, was associated with greater alcohol consumption (Study 10). Another study found that impulsivity when distressed partially explained the relationship between PTSD and substance use for women, but this relationship was partially explained by emotional awareness/clarity for men (Study 1). A third study found that difficulties with goal-directed behavior when distressed above and beyond other emotion regulation abilities had a full indirect effect in the association between avoidance and numbing PTSD symptoms and alcohol dependence (Study 2).

In comparison, four studies did not find significant indirect effects of difficulties regulating negative emotions in the PTSD-substance use association (Studies 3, 5, 6, and 11). Nonetheless, one of these studies reported that emotion regulation difficulties had an indirect effect on the association between PTSD and alcohol use coping motives. This suggested that individuals with PTSD experienced increased emotion regulation difficulties and were subsequently at increased risk of using alcohol to cope with negative emotions (Study 6). Furthermore, when considering men only, one of these studies found that impulsivity when distressed and a lack of emotional clarity had an indirect effect on the PTSD-substance use association (Study 11).

In addition, four studies found difficulties regulating positive emotions had a significant indirect effect on the association between PTSD and substance use (Studies 3, 12–14). In one of these studies alcohol use to regulate negative emotions (despondency and anger) and positive emotions had an indirect effect on the association between PTSD and alcohol use, separately. When examining alcohol use to regulate positive and negative emotions simultaneously, only alcohol use to regulate despondency made a significant and unique contribution to the association between PTSD and alcohol use (Study 13). Furthermore, one study demonstrated significant indirect effects in both directions, i.e., substance use also had an indirect effect on the association between PTSD and positive emotion dysregulation, suggesting bidirectional relationships (Study 14). One of these studies focused specifically on emotional avoidance demonstrating that the avoidance of positive emotions, but not the intensity of positive emotions, had a significant indirect effect on the association between PTSD symptoms and alcohol use. In a sequential model, positive emotional intensity was associated with greater positive emotional avoidance and this sequential relationship indirectly effected the association between PTSD and alcohol use (Study 12).

#### Moderation Studies of Emotion Regulation Abilities

One study considered emotion regulation (and coping self-efficacy) as moderators in the association between PTSD severity and drug use, in a sample of women with experience of sexual assault (Study 17). For women with less emotion regulation difficulties and high coping self-efficacy, there was no significant relationship between PTSD and drug use, indicating they might act as protective factors in this association.

#### Group Comparison Studies of Emotion Regulation Abilities

Five studies considered between-group differences in emotion regulation (Studies 18–22). Three studies with clinical samples demonstrated significantly increased difficulties with emotion regulation among PTSD-SUD patients compared to SUD-only patients (Studies 19, 21, and 22). In two of these studies, PTSD-SUD patients reported significantly greater emotion regulation difficulties across all abilities, except emotional awareness (Studies 19 and 21). Furthermore, in these studies, emotion regulation was a unique predictor of PTSD status above and beyond other mechanisms examined, including negative urgency, sensation seeking, and anxiety sensitivity, suggesting that it may be an important explanatory mechanism (Studies 19 and 21). In the third study, participants with PTSD-SUD versus SUD alone reported significantly more difficulties with goal-directed behavior when experiencing negative emotions, impulsivity when distressed, and limited access to emotion regulation strategies. Further analyses revealed impulsivity when distressed was the only dimension uniquely associated with probable PTSD status (Study 22).

Two studies also included participants with PTSD only (Studies 18 and 20). One study found firefighters with PTSD and Alcohol Use Disorder (AUD) reported significantly greater difficulties with emotion regulation than firefighters with trauma exposure or AUD only, but not PTSD alone (Study 18). Similarly, in another clinical sample, the highest levels of emotion dysregulation were observed among participants with PTSD only, compared to those with PTSD-SUD, or SUD (Study 20). However, in an additional exploratory question, the PTSD-SUD group was significantly more likely to report using substances to feel less emotion than the SUD-only group.

#### Cluster and Latent Subgroup Studies of Emotion Regulation Abilities

Three studies examined patterns of emotion regulation across different groups based on cluster and latent profile analyses (Studies 24–26). Two of these studies looked at difficulties regulating negative emotions (Studies 24 and 26). One study identified three groups in their cluster analysis: clinically distressed (clinical levels of PTSD, probable substance dependence), subclinical distress with alcohol use (subclinical PTSD symptoms, early to middle problem drinking), and subclinical distress without alcohol use (subclinical PTSD symptoms, significantly lower alcohol use) (Study 24). The clinically distressed group reported significantly more difficulties with emotion regulation than both other groups, while the subclinical distress group without alcohol use reported the least emotion regulation difficulties. Non-acceptance of emotions and impulsivity when distressed were significant predictors of group membership, specifically distinguishing between the groups without alcohol use and the groups with alcohol use. The clinically distressed group showed significantly more difficulties on all dimensions of emotion regulation, except awareness.

Furthermore, another study identified four latent profiles of individuals based on their experiences of trauma, PTSD symptoms, and emotion regulation, and then considered group differences in alcohol use (Study 26). Two out of four profiles were characterized by PTSD symptoms and significant deficits in emotion regulation, except for awareness in one profile. These profiles demonstrated elevated drinking frequency and quantity when compared with the other profiles which were characterized by lower levels of emotion regulation difficulties. The third study considered difficulties regulating positive and negative emotions among women with experience of domestic violence (Study 25). The group characterized by difficulties regulating both positive and negative emotions (vs. low difficulty with emotion regulation) reported significantly more severe PTSD symptoms and alcohol use. The results approached significance for drug use. There were no significant differences between groups characterized by difficulties regulating both positive and negative emotions or negative emotions only.

#### Other Cross-Sectional Studies of Emotion Regulation Abilities

Two studies considered associations between emotion regulation abilities, PTSD, and alcohol use (Studies 27 and 28). One study found that emotion regulation difficulties significantly predicted PTSD and alcohol dependence among a clinical sample (Study 27). This study accounted for comorbidity by controlling for the interrelationships between PTSD, alcohol use, depression, anxiety, and insomnia. These findings suggest that emotion regulation difficulties are a transdiagnostic construct that uniquely predicts PTSD and alcohol use severity. When considering specific emotion regulation abilities, this study excluded the DERS awareness subscale but demonstrated that none of the other DERS subscales was significantly related to alcohol dependence, while controlling for PTSD (and insomnia, anxiety, and depression). However, difficulties with goal-directed behavior when distressed and impulsivity when distressed were significantly associated with PTSD symptom severity. The other study found that among a veteran sample, PTSD, but not alcohol use, significantly predicted emotion regulation difficulties, including total DERS scores and all subscales, except awareness (Study 28). These results highlight specific emotion regulation abilities which may be associated with PTSD, but in these studies were not related to alcohol use.

#### Prospective Longitudinal Studies of Emotion Regulation Abilities

Two prospective longitudinal studies considered emotion regulation in the PTSD-substance use association (Studies 30 and 31). One study demonstrated a significant positive association between PTSD symptoms at baseline and substance use 8 months later, only for participants with increased difficulties with emotion regulation at baseline (Study 30). When considering specific abilities, there was a significant positive association between PTSD symptoms at baseline and substance use 8 months later, only for participants who indicated low willingness to experience distress to pursue goals. Meanwhile, in a study of women experiencing intimate partner violence, in which momentary assessments were conducted three times a day over 30 days, PTSD moderated the association between difficulties regulating positive emotions and drug, but not alcohol, use. PTSD did not moderate the association between difficulties regulating negative emotions and substance use. These findings suggest that those experiencing intimate partner violence may be at an increased risk of substance use in response to positive and negative emotions, regardless of their PTSD status (Study 31).

#### Secondary Analyses of RCTs Considering Emotion Regulation Abilities

Two secondary analyses of RCTs examined emotion regulation during treatment for co-occurring PTSD and SUDs (Study 32) and co-occurring depression and alcohol dependence (Study 33). One study demonstrated the moderating role of emotion regulation in psychological therapies for PTSD and substance use outcomes. Individuals with high levels of emotion dysregulation at baseline demonstrated greater reductions in PTSD symptoms when they received a trauma-focused intervention, compared with a substance use focused intervention. In contrast, when considering substance use outcomes, those with low emotion dysregulation benefited more from addiction-focused treatments than those with high emotion dysregulation. These findings suggest treatment optimization based on levels of emotion regulation may be useful (Study 32). The other study examined whether changes in emotion regulation during treatment predicted depression and PTSD symptom severity at treatment completion and subsequent alcohol use. Findings indicated that lower depression at the end of treatment, but not PTSD symptoms, mediated the association between improvements in emotion regulation during treatment and greater abstinence following treatment. Further analyses indicated that it was changes in emotion regulation which led to reduced drinking through reduced depression symptoms and not that better emotion regulation led to reduced alcohol use and subsequent improvements in depression (Study 33).

#### Emotion Regulation Strategies

Three studies looked at emotion regulation strategies, cognitive reappraisal, and expressive suppression (Studies 16, 23, and 29). Two of these studies were conducted with military samples (Studies 16 and 29) and one in a community sample (Study 23). All three studies focused on alcohol use. Findings regarding the role of these emotion regulation strategies were mixed. One study demonstrated a significant positive association between PTSD symptoms and alcohol use only in the context of low baseline cognitive reappraisal, such that those with more severe PTSD symptoms tended to use more alcohol over 1 year when they exhibited low levels of cognitive reappraisal at baseline (Study 29). In contrast, another study found cognitive reappraisal moderated the association between PTSD symptoms and AUD, such that there was a stronger association among individuals with high levels of cognitive reappraisal (Study 16). Finally, in a latent profile analysis, cognitive reappraisal did not differ among the profiles (low PTSD-AUD, mild PTSD-moderate AUD, moderate PTSD-low AUD, and high PTSD-AUD) with the overall sample exhibiting levels of cognitive reappraisal similar to that observed in other studies of PTSD (Study 23).

Mixed results were also demonstrated when considering expressive suppression. Two studies found increased expressive suppression to be related to PTSD and alcohol use (Studies 16 and 23). One study demonstrated the moderating role of expressive suppression in the association between PTSD symptoms and alcohol use, with a stronger association observed in individuals exhibiting high levels of expressive suppression (Study 16). Expressive suppression was also elevated in more severe PTSD/AUD latent profiles and was significantly different between low PTSD and moderate/high PTSD profiles suggesting it may be particularly related to PTSD symptom severity (Study 23). However, this relationship was not observed in a third study which found no main effect of expressive suppression in the association between PTSD and alcohol use and no interaction effects between expressive suppression and PTSD when predicting alcohol use over 1 year (Study 29).

#### Trauma Type, Trauma Exposure, and Emotion Regulation in PTSD and Substance Use

Findings from studies of different trauma types (military trauma, intimate partner violence, childhood and adolescent trauma, and sexual assault) demonstrated a significant role for difficulties regulating emotions in the PTSD-substance use association, suggesting this relationship is consistent across exposure to different types of traumas (Studies 4, 11, 16, 17, 22, 24, 25, 29 and 31). Twelve studies considered trauma type as a potential covariate in their analyses (Studies 2, 5–9, 12, 13, 16, 17, 19, and 29), but a limited number specifically examined trauma exposure, or compared trauma types. One study with a military sample, demonstrated no correlations between trauma severity and PTSD symptoms, emotion dysregulation or alcohol use, suggesting these variables were not related (Study 28). However, two studies with clinical samples found PTSD-SUD patients reported the greatest trauma severity, when compared to SUD or PTSD alone. PTSD-SUD patients also reported greater levels of emotion dysregulation when compared to SUD, but not PTSD, only groups (Studies 20 and 22). Furthermore, one study found physical abuse was associated with low impulse control and access to emotion regulation strategies, whereas emotional abuse was associated with all emotion regulation difficulties (Study 22). Furthermore, another study found child and adolescent sexual abuse, rather than cumulative trauma, was more strongly related to emotion regulation difficulties among college students, suggesting this type of trauma may have a stronger effect on emotion regulation (Study 4).

Two studies considered group variation in trauma exposure, PTSD, alcohol use, and emotion regulation, so highlighting differential presentations across these domains (Studies 24 and 26). One study found those with experience of multiple traumas who reported the highest likelihood of experiencing PTSD symptoms had elevated emotion regulation difficulties and the highest levels of problematic drinking, compared to groups characterized by low levels of emotion regulation difficulties. However, this group experienced similar levels of alcohol-related problems to another group who reported PTSD symptoms and elevated emotion regulation difficulties, but a lower probability of cumulative trauma. This may suggest that PTSD symptoms and emotion regulation, rather than cumulative trauma may be risk factors for alcohol use (Study 26). Furthermore, another study found the highest levels of interpersonal trauma exposure among a clinically distressed group (clinical levels of PTSD, probable substance dependence), who also reported the highest levels of emotion dysregulation compared with subclinical distress groups (subclinical PTSD symptoms, early to middle problem drinking or low alcohol use). However, the subclinical distress without alcohol use group reported the next highest level of interpersonal trauma, with no significant difference when compared to the clinical distress group (Study 24).

#### Gender and Emotion Regulation in PTSD and Substance Use

Of the included studies, seven focused specifically on women (Studies 2, 17, 24, 25, 30, 31, and 33) and two on men (Studies 5 and 16). These studies largely demonstrated a significant role for emotion regulation in the PTSD-substance use association, suggesting that this association is observed across men and women. Gender or sex at birth was controlled for in twelve studies, with significant results remaining consistent (Studies 6, 8–10, 13–15, 20–23, and 26). One study also examined whether the models they tested fit equally well for men and women (Study 4). The results suggested the observed patterns in emotion regulation remained constant when looking at the overall sample and when allowing for variability by gender. Together, these results suggest robust findings beyond the effects of gender. However, the role of gender is complex and studies focusing specifically on gender identified more nuanced relationships with emotion regulation.

Only two studies specifically examined gender differences in emotion regulation (Studies 1 and 3). One study with a clinical sample, examined impulsivity when distressed and lack of emotional awareness and clarity in the association between PTSD and alcohol and drug use (Study 1). The results demonstrated that for women the association between PTSD and substance use frequency was partially explained by impulsivity when distressed whereas for men, this association was partially explained by emotional awareness and clarity. PTSD symptoms were consistently associated with impulsivity when distressed and awareness and clarity across genders, suggesting that these gender differences are specific to the PTSD-substance use association. Moreover, another study found difficulties regulating positive emotions had a significant indirect effect in the association between PTSD symptom severity for men but not for women (Study 3).

#### Social Factors and Emotion Regulation in PTSD and Substance Use

All the included studies measured some social factors, including employment, education, income, and relationship status, although none of the included studies collected data on housing. Seven studies included social factors as covariates in their final analyses (Studies 5, 6, 8, 9 16, 18, and 31). Relationship status was included in five of these studies as a covariate (Studies 5, 9 16, 18, and 31). For example, one study found that trauma-exposed only participants, compared to those with probable PTSD and/or probable AUD, were significantly more likely to be in a current romantic relationship (Study 18). Another study demonstrated a positive association between emotion regulation difficulties at baseline with alcohol-related interpersonal consequences, drinking frequency, and severity of PTSD symptoms (Study 33). The authors suggested that emotion regulation difficulties may contribute to PTSD symptoms and alcohol use and that there may be a cyclical effect in which emotion dysregulation leads to difficulties managing interpersonal relationships which perpetuates alcohol use and vice versa.

One study included a specific measure of social adjustment, considering functioning across a range of areas including work, social and leisure activities, relationships, and parental and family roles. Individuals with PTSD (vs. combat exposed only and unexposed to combat) reported lower social adjustment and demonstrated significantly more difficulties with emotion regulation and alcohol use. Differences in social adjustment scores approached statistical significance, and therefore warrant further exploration (Study 5). Finally, one study examined occupational duration and stress in a sample of firefighters, finding that both were positively and significantly related to PTSD symptom severity, emotion regulation difficulties, and alcohol use, suggesting employment-related factors may influence these variables (Study 6).

### Discussion

This review examined studies investigating the role of emotion regulation in the PTSD-substance use association and brings together a body of heterogenous research, synthesizing our understanding of this complex topic. Findings highlight the importance of emotion regulation in the development, maintenance, and treatment of PTSD-substance use. Specifically, while there were some mixed findings in studies of indirect effects, eleven out of fifteen studies found PTSD was associated with difficulties regulating negative and/or positive emotions and, in turn, increased substance use. Possible explanations for the contradictory results from some studies include small sample sizes underpowered to detect significant results, a focus on specific populations including military personnel and firefighters, and the exclusion of individuals with alcohol dependence. Studies also suggested individuals with PTSD-SUD compared to SUD only and individuals with more severe PTSD and substance use symptoms experience increased difficulties with emotion regulation. While a limited number of longitudinal studies were identified for review, available findings suggested that emotion regulation difficulties were associated with an elevated risk of substance use over time, and emotion regulation was shown to influence PTSD and substance use outcomes during treatment. These findings require replication.

Among clinical samples, broad deficits in emotion regulation abilities were observed. Several studies highlighted impulsivity when distressed and a lack of goal-directed behavior when distressed as particularly relevant to the PTSD-substance use association. However, a limited number of studies examined specific emotion regulation abilities, and this requires further exploration. Despite this, the findings are consistent with extant literature suggesting behavioral emotion regulation abilities such as impulsivity when distressed, are more strongly related to substance use (Weiss, Kiefer, et al., 2021). In contrast, emotional awareness was largely not significant in the PTSD-substance use association, which may reflect the suggestion that this represents a distinct underlying construct (Bardeen et al., 2012). Only a small number of studies examined emotion regulation strategies, producing mixed findings and highlighting the need for further studies in this area. One possible explanation for these contradictory findings is the context-dependent nature of emotion regulation, such that the use of these strategies may be maladaptive or adaptive depending on the situation (Aldao et al., 2010; Christ et al., 2022; Wen et al., 2024). Capturing context has been highlighted as an important avenue for further research as the study of emotion regulation progresses (Petrova & Gross, 2023).

The review also sought to examine the contributions of gender, trauma type, and social factors to emotion regulation in the PTSD-substance use association. Findings are preliminary but suggest there may be heterogeneity in the specific emotion regulation abilities explaining the PTSD-substance use association for men and women. This is reflective of wider literature demonstrating gender differences in emotion regulation (Delhom et al., 2021). Although there was limited consideration of trauma type, experiences of trauma may be differentially associated with emotion regulation (Klanecky et al., 2016; Weiss, Tull, Lavender, et al., 2013). Given existing evidence suggests interpersonal and cumulative trauma may be particularly associated with deficits in emotion regulation, this area warrants further investigation (Ehring & Quack, 2010). Examination of social factors was also limited. However, relationships were highlighted as relevant. Relationships may act as a protective factor in the development of PTSD and substance use, or those with PTSD-substance use difficulties may be less likely to maintain relationships (Lebeaut et al., 2021). Both conceptualizations are consistent with literature suggesting that social support is associated with resilience to PTSD following trauma exposure and that PTSD may reduce and compromise social support resources (Wang et al., 2021).

The synthesis presented adds to our theoretical understanding of the PTSD-substance use association and seems largely consistent with the self-medication hypothesis and negative reinforcement models, in which the functional role of substance use is to relieve negative affect, for example, PTSD-related distress (Baker et al., 2004; Khantzian, 1997). Furthermore, findings support the idea that difficulties regulating positive emotions may be important in the PTSD-substance use association. It has been suggested that positive emotions may be experienced as aversive or, alternatively, substance use may be an attempt to enhance or maintain a positive emotional state (Goncharenko et al., 2019; Litz et al., 2000; Weiss et al., 2019). As such, it is possible that both positive and negative reinforcement processes are important in the PTSD-substance use association, and the examination of motivations for substance use in the context of positive and negative emotions warrants further exploration.

The findings from this review should be interpreted in light of some limitations. Firstly, there was large heterogeneity in the included studies which precluded meta-analysis and direct comparisons between studies. Moreover, the included studies were characterized by small self-selecting samples and predominantly used self-report measures of emotion regulation, substance use, and PTSD which may have introduced bias and failed to capture the complexity and nuance of emotional experience. Relatedly, despite the scope of this review, no qualitative studies were identified. The cross-sectional nature of most studies also largely precluded temporal and causal conclusions. In addition, most studies considered the relationship between the study variables in one direction, i.e., the effects of PTSD via emotion dysregulation on substance use, making it difficult to draw conclusions about directionality.

Furthermore, most of the reviewed studies focused on alcohol use. Evidence suggests that different substances may show different relationships with emotion regulation (e.g., polysubstance use may be particularly associated with difficulties regulating emotions) (Weiss, Kiefer, et al., 2021). Meanwhile, studies that focused on concepts related to, but distinct from substance use (e.g., substance use expectancies, substance use motives, and cravings) were excluded. These elements of substance use may have unique relationships with PTSD and emotion regulation (Holzhauer et al., 2024). Other factors which may be relevant to this association, including ethnicity and psychiatric comorbidities, were not considered. Racial and ethnic differences in emotion regulation have been observed across various studies (Weiss, Thomas, et al., 2022), and emotion regulation has been associated with a range of mental health difficulties, including anxiety and depression, which commonly cooccur with PTSD and substance use (Sloan et al., 2017). In addition, gender and sex were largely conflated in the included studies and it was often unclear how “gender” was determined, precluding generalisability and applications to clinical practice. Moreover, none of the included studies focused on gender minorities, who tend to experience high rates of trauma, PTSD, and substance use (Brewerton et al., 2022; Parent et al., 2019).

This review highlights important avenues for future research. First, studies with larger samples are needed, possibly using multi-modal assessment of study variables, to reduce bias and improve generalisability. Prospective longitudinal studies could also shed light on causality. Replication of findings on gender, in which gender is clearly measured and reported (see SAGER guidelines; Heidari et al.,2016) is required, along with the examination of emotion regulation in gender minorities experiencing PTSD and substance use. Research should also consider emotion regulation in relation to concepts related to substance use, for example, craving, racial and ethnic differences, and other psychiatric comorbidities. Studies comparing different trauma types and substances are needed. An exploration of how social factors influence emotion regulation, along with the examination of the context in which emotion regulation occurs is warranted. Importantly, qualitative work could offer a new perspective, situating emotion regulation in the context of individuals’ lives and social circumstances while providing more in-depth insights into lived and living experiences. This could support a more nuanced understanding of how emotion regulation relates to trauma, PTSD, and substance use.

Turning to clinical implications, emotion regulation may represent an important integrated treatment target. This is consistent with current UK guidelines which highlight the possible benefits to service users of offering concurrent support for substance use and PTSD (NICE, 2018; Office for Health Improvement & Disparities, 2023). Treatment with a specific focus on emotion regulation, such as Dialectical Behavior Therapy (DBT), may be useful in enhancing emotion regulation skills in PTSD-SUD populations. DBT has shown promising results in reducing PTSD symptoms and substance use, and in improving emotion regulation skills (Bohus et al., 2020; Warner & Murphy, 2022). Moreover, assessment and treatment which focuses on the specific emotion regulation difficulties experienced by an individual may likewise be beneficial. When exploring the connections between PTSD and substance use, difficulties regulating both positive and negative emotions should be considered in treatment protocols. While further research is needed, adjustments based on gender and trauma type may be beneficial along with support with social factors such as maintaining healthy relationships. Evaluation of the impact of such treatment and assessment efforts, alongside patients’ experiences, represents an important avenue for future research.

To conclude, this review adds to our understanding of the relationship between PTSD and substance use by demonstrating the role of difficulties regulating negative and positive emotions in this comorbidity. Findings highlight specific emotion regulation difficulties which may be particularly relevant. Moreover, there may be differences in emotion regulation in the PTSD-substance use association based on gender, trauma type, and social factors, though this requires further examination. Directions for future research include longitudinal studies to delineate causation, the examination of emotion regulation among gender minorities, racial and ethnic differences, and context and lived experience which may be achieved through qualitative research. The results have clinical implications for the assessment and treatment of PTSD-substance use, with integrated treatment efforts potentially benefiting from focusing on improving emotion regulation skills.

## Supplemental Material

sj-docx-1-tva-10.1177_15248380241306362 – Supplemental material for Emotion Regulation in the Association Between Posttraumatic Stress Disorder and Substance Use: A Systematic Review With Narrative Synthesis Supplemental material, sj-docx-1-tva-10.1177_15248380241306362 for Emotion Regulation in the Association Between Posttraumatic Stress Disorder and Substance Use: A Systematic Review With Narrative Synthesis by Alice May Bowen, Robert Calder, Joanne Neale, Tim Meynen and Gail Gilchrist in Trauma, Violence, & Abuse

sj-docx-2-tva-10.1177_15248380241306362 – Supplemental material for Emotion Regulation in the Association Between Posttraumatic Stress Disorder and Substance Use: A Systematic Review With Narrative Synthesis Supplemental material, sj-docx-2-tva-10.1177_15248380241306362 for Emotion Regulation in the Association Between Posttraumatic Stress Disorder and Substance Use: A Systematic Review With Narrative Synthesis by Alice May Bowen, Robert Calder, Joanne Neale, Tim Meynen and Gail Gilchrist in Trauma, Violence, & Abuse

sj-docx-3-tva-10.1177_15248380241306362 – Supplemental material for Emotion Regulation in the Association Between Posttraumatic Stress Disorder and Substance Use: A Systematic Review With Narrative Synthesis Supplemental material, sj-docx-3-tva-10.1177_15248380241306362 for Emotion Regulation in the Association Between Posttraumatic Stress Disorder and Substance Use: A Systematic Review With Narrative Synthesis by Alice May Bowen, Robert Calder, Joanne Neale, Tim Meynen and Gail Gilchrist in Trauma, Violence, & Abuse

sj-docx-4-tva-10.1177_15248380241306362 – Supplemental material for Emotion Regulation in the Association Between Posttraumatic Stress Disorder and Substance Use: A Systematic Review With Narrative Synthesis Supplemental material, sj-docx-4-tva-10.1177_15248380241306362 for Emotion Regulation in the Association Between Posttraumatic Stress Disorder and Substance Use: A Systematic Review With Narrative Synthesis by Alice May Bowen, Robert Calder, Joanne Neale, Tim Meynen and Gail Gilchrist in Trauma, Violence, & Abuse

## References

[bibr1-15248380241306362] Aase D. M. Gorka S. M. Greenstein J. E. Proescher E. Crane N. A. Everett L.K. Hassan I. Osborn A. Schroth C. Kennedy-Krage A. Phan K. (2018). Cognitive reappraisal moderates the relationship between PTSD symptoms and alcohol use over time in post-9/11 U.S. military veterans. Drug and Alcohol Dependence, 191, 159–164. 10.1016/j.drugalcdep.2018.06.032. 30118943

[bibr2-15248380241306362] Aldao A. Nolen-Hoeksema S. Schweizer S. (2010). Emotion-regulation strategies across psychopathology: A meta-analytic review. Clinical Psychology Review, 30(2), 217–237. 10.1016/j.cpr.2009.11.004 20015584

[bibr3-15248380241306362] American Psychiatric Association (APA). (1980). Diagnostic and Statistical Manual of Mental Disorders (Vol. 3rd).

[bibr4-15248380241306362] Baker T. B. Piper M. E. McCarthy D. E. Majeskie M. R. Fiore M. C. (2004). Addiction motivation reformulated: An affective processing model of negative reinforcement. Psychological Review, 111(1), 33–51. 10.1037/0033-295x.111.1.33 14756584

[bibr5-15248380241306362] Bardeen J. R. Fergus T. A. Orcutt H. K. (2012). An examination of the latent structure of the difficulties in emotion regulation scale. Journal of Psychopathology and Behavioral Assessment, 34(3), 382–392. 10.1007/s10862-012-9280-y

[bibr6-15248380241306362] Berking M. Margraf M. Ebert D. Wupperman P. Hofmann S. G. Junghanns K. (2011). Deficits in emotion-regulation skills predict alcohol use during and after cognitive-behavioral therapy for alcohol dependence. Journal of Consulting & Clinical Psychology, 79(3), 307–318. 10.1037/a0023421 21534653 PMC3109184

[bibr7-15248380241306362] Bohus M. Kleindienst N. Hahn C. Müller-Engelmann M. Ludäscher P. Steil R. Fydrich T. Kuehner C. Resick P. A. Stiglmayr C. Schmahl C. Priebe K. (2020). Dialectical behavior therapy for posttraumatic stress disorder (DBT-PTSD) compared with cognitive processing therapy (CPT) in complex presentations of PTSD in women survivors of childhood abuse. JAMA Psychiatry, 77(12), 1235. 10.1001/jamapsychiatry.2020.2148 PMC7376475 32697288

[bibr8-15248380241306362] Bornovalova M. A. Ouimette P. Crawford A. V. Levy R. (2009). Testing gender effects on the mechanisms explaining the association between post-traumatic stress symptoms and substance use frequency. Addictive Behaviors, 34(8), 685–692. 10.1016/j.addbeh.2009.04.005 19423233 PMC2746089

[bibr9-15248380241306362] Brady K. T. Back S. E. Coffey S. F. (2004). Substance abuse and posttraumatic stress disorder. Current Directions in Psychological Science, 13(5), 206–209. 10.1111/j.0963-7214.2004.00309.x

[bibr10-15248380241306362] Brewerton T. D. Suro G. Gavidia I. Perlman M. M. (2022). Sexual and gender minority individuals report higher rates of lifetime traumas and current PTSD than cisgender heterosexual individuals admitted to residential eating disorder treatment. Eating and Weight Disorders, 27(2), 813–820. 10.1007/s40519-021-01222-4 34057704

[bibr11-15248380241306362] Chilcoat H. D. Breslau N. (1998). Investigations of causal pathways between PTSD and drug use disorders. Addictive Behaviors, 23(6), 827–840. 10.1016/s0306-4603(98)00069-0 9801719

[bibr12-15248380241306362] Christ N. M. Byllesby B. M. Elhai J. D. (2022). The effect of cognitive-affective factors on PTSD and alcohol use symptoms: An investigation on rumination, suppression, and reappraisal. Substance Use & Misuse, 57(14), 2053–2062. 10.1080/10826084.2022.2129997 36305851

[bibr13-15248380241306362] Currie C. L. (2021). Adult PTSD symptoms and substance use during Wave 1 of the COVID-19 pandemic. Addictive Behaviors Reports, 13, 100341. 10.1016/j.abrep.2021.100341 33763517 PMC7973858

[bibr14-15248380241306362] Delhom I. Melendez J. C. Satorres E. (2021). The regulation of emotions: Gender differences. European Psychiatry, 64(S1), S836–S836. 10.1192/j.eurpsy.2021.2209

[bibr15-15248380241306362] Dixon-Gordon K. L. Tull M. T. Gratz K. L. (2014). Self-injurious behaviors in posttraumatic stress disorder: An examination of potential moderators. Journal of Affective Disorders, 166, 359–367. 10.1016/j.jad.2014.05.033 24981133 PMC4155484

[bibr16-15248380241306362] Drapkin M. L. Yusko D. Yasinski C. Oslin D. Hembree E. A. Foa E. B. (2011). Baseline functioning among individuals with posttraumatic stress disorder and alcohol dependence. Journal of Substance Abuse Treatment, 41(2), 186–192. 10.1016/j.jsat.2011.02.012 21546205 PMC3144264

[bibr17-15248380241306362] Ehlers A. Clark D. M. (2000). A cognitive model of posttraumatic stress disorder. Behaviour Research & Therapy, 38(4), 319–345. 10.1016/s0005-7967(99)00123-0 10761279

[bibr18-15248380241306362] Ehring T. Quack D. (2010). Emotion regulation difficulties in trauma survivors: The role of trauma type and PTSD symptom severity. Behavior Therapy, 41(4), 587–598. 10.1016/j.beth.2010.04.004 21035621

[bibr19-15248380241306362] English T. John O. P. Gross J. J. (2013). Emotion regulation in close relationships. In Simpson J. A. Campbell L. (Eds.), The Oxford handbook of close relationships. Oxford Library of Psychology. 10.1093/oxfordhb/9780195398694.013.0022

[bibr20-15248380241306362] English T. Lee I. A. John O. P. Gross J. J. (2017). Emotion regulation strategy selection in daily life: The role of social context and goals. Motivation & Emotion, 41(2), 230–242. 10.1007/s11031-016-9597-z 28652647 PMC5482525

[bibr21-15248380241306362] Fairholme C. P. Nosen E. L. Nillni Y. I. Schumacher J. A. Tull M. T. Coffey S. F. (2013). Sleep disturbance and emotion dysregulation as transdiagnostic processes in a comorbid sample. Behaviour Research and Therapy, 51(9), 540–546. 10.1016/j.brat.2013.05.014 23831496 PMC3774794

[bibr22-15248380241306362] Fallot R. D. Harris M. (2005). Integrated trauma services teams for women survivors with alcohol and other drug problems and co-occurring mental disorders. Alcoholism Treatment Quarterly, 22(3–4), 181–199. 10.1300/J020v22n03_10

[bibr23-15248380241306362] Feingold D. Zerach G. (2021). Emotion regulation and experiential avoidance moderate the association between posttraumatic symptoms and alcohol use disorder among Israeli combat veterans. Addictive Behaviors, 115, 106776. 10.1016/j.addbeh.2020.106776 33348279

[bibr24-15248380241306362] Garke M. A. Isacsson N. H. Sorman K. Bjureberg J. Hellner C. Gratz K. L. Berghoff C. R. Sinha R. Tull M. T. Jayaram-Lindstrom N. (2021). Emotion dysregulation across levels of substance use. Psychiatry Research, 296, 113662. 10.1016/j.psychres.2020.113662. 33406445

[bibr25-15248380241306362] Goldstein B. Bradley B. Ressler K. J. Powers A. (2017). Associations between posttraumatic stress disorder, emotion dysregulation, and alcohol dependence symptoms among inner city females. Journal of Clinical Psychology, 73(3), 319–330. 10.1002/jclp.22332 27467499 PMC5324595

[bibr26-15248380241306362] Goncharenko S. Weiss N. H. Contractor A. A. Dixon-Gordon K. L. Forkus S. R. (2019). The role of gender in the associations among posttraumatic stress disorder symptom, severity, difficulties regulating emotions, and alcohol misuse. Addictive Behaviors, 99, 106086. 10.1016/j.addbeh.2019.106086 31445483 PMC6791770

[bibr27-15248380241306362] Gratz K. L. Roemer L. (2004). Multidimensional assessment of emotion regulation and dysregulation: Development, factor structure, and initial validation of the difficulties in emotion regulation scale. Journal of Psychopathology and Behavioral Assessment, 26(1), 41–54. 10.1023/B:JOBA.0000007455.08539.94

[bibr28-15248380241306362] Greenberg N. Brooks S. Dunn R. (2015). Latest developments in post-traumatic stress disorder: Diagnosis and treatment. British Medical Bulletin, 114(1), 147–155. 10.1093/bmb/ldv014 25904382

[bibr29-15248380241306362] Gross J. J. (1998). The emerging field of emotion regulation: An integrative review. Review of General Psychology, 2(3), 271–299. 10.1037/1089-2680.2.3.271

[bibr30-15248380241306362] Gross J. J. John O. P. (2003). Individual differences in two emotion regulation processes: Implications for affect, relationships, and well-being. Journal of Personality and Social Psychology, 85(2), 348–362. 10.1037/0022-3514.85.2.348 12916575

[bibr31-15248380241306362] Gross J. J. (2015). The extended process model of emotion regulation: Elaborations, applications, and future directions. Psychological Inquiry, 26(1), 130–137. 10.1080/1047840X.2015.989751

[bibr32-15248380241306362] Haller M. Chassin L. (2013). The influence of PTSD symptoms on alcohol and drug problems: Internalizing and externalizing pathways. Psychological Trauma: Theory, Research, Practice, and Policy, 5, 484–493. 10.1037/a0029335

[bibr33-15248380241306362] Hawn S. E. Cusack S. E. Amstadter A. B. (2020). A systematic review of the self-medication hypothesis in the context of posttraumatic stress disorder and comorbid problematic alcohol use. Journal of Traumatic Stress, 33(5), 699–708. 10.1002/jts.22521 32516487 PMC7572615

[bibr34-15248380241306362] Heidari S. Babor T. F. De Castro P. Tort S. Curno M. (2016). Sex and gender equity in research: Rationale for the SAGER guidelines and recommended use. Research Integrity and Peer Review, 1(1). 10.1186/s41073-016-0007-6 PMC5793986 29451543

[bibr35-15248380241306362] Hien D. A. Lopez-Castro T. Papini S. Gorman B. Ruglass L. M. (2017). Emotion dysregulation moderates the effect of cognitive behavior therapy with prolonged exposure for co-occurring PTSD and substance use disorders. Journal of Anxiety Disorders, 52, 53–61. 10.1016/j.janxdis.2017.10.003 29049902 PMC5728385

[bibr36-15248380241306362] Holzhauer C. G. Gamble S. A. (2017). Depressive symptoms mediate the relationship between changes in emotion regulation during treatment and abstinence among women with alcohol use disorders. Psychology of Addictive Behaviors, 31(3), 284. 10.1037/adb0000274 28368158 PMC5431747

[bibr37-15248380241306362] Holzhauer C. G. Sherrill A. Musicaro R. M. Ellis R. A. (2024). The Role of Emotion Dysregulation in heightened alcohol craving related to posttraumatic stress disorder and depression symptoms. Substance Use & Misuse, 59(6), 874–885. 10.1080/10826084.2024.2305805 38263678

[bibr38-15248380241306362] Hong Q. N. Fàbregues S. Bartlett G. Boardman F. Cargo M. Dagenais P. Gagnon M.-P. Griffiths F. Nicolau B. O’Cathain A. Rousseau M.-C. Vedel I. Pluye P. (2018). The Mixed Methods Appraisal Tool (MMAT) version 2018 for information professionals and researchers. Education for Information, 34, 285–291. 10.3233/EFI-180221

[bibr39-15248380241306362] Kahl J. Holl J. Grundmann J. Lotzin A. Hiller P. Schroeder K. Schulte B. Barnow S. Schäfer I. (2020). Emotion regulation as a mediator between childhood abuse and neglect and posttraumatic stress disorder in women with substance use disorders. Substance Use & Misuse, 55(13), 2184–2193. 10.1080/10826084.2020.1797805 32835585

[bibr40-15248380241306362] Kaysen D. Atkins D. C. Moore S. A. Lindgren K. P. Dillworth T. Simpson T. (2011). Alcohol use, problems, and the course of posttraumatic stress disorder: A prospective study of female crime victims. Journal of Dual Diagnosis, 7(4), 262–279. 10.1080/15504263.2011.620449 23538605 PMC3607458

[bibr41-15248380241306362] Khantzian E. J. (1997). The self-medication hypothesis of substance use disorders: A reconsideration and recent applications. Harvard Review of Psychiatry, 4(5), 231–244. 10.3109/10673229709030550 9385000

[bibr42-15248380241306362] Klanecky A. K. McChargue D. E. Tuliao A. P. (2016). Proposed pathways to problematic drinking via post-traumatic stress disorder symptoms, emotion dysregulation, and dissociative tendencies following child/adolescent sexual abuse. Journal of Addictive Disorders, 35(3), 180–193. 10.1080/10550887.2016.1139428 26756960

[bibr43-15248380241306362] Klemanski D. H. Mennin D. S. Borelli J. L. Morrissey P. M. Aikins D. E. (2012). Emotion-related regulatory difficulties contribute to negative psychological outcomes in active-duty iraq war soldiers with and without posttraumatic stress disorder. Depression & Anxiety (1091–4269), 29(7), 621–628. 10.1002/da.21914 22461455

[bibr44-15248380241306362] Lebeaut A. Zegel M. Leonard S. J. Bartlett B. A. Vujanovic A. A. (2021). Examining transdiagnostic factors among firefighters in relation to trauma exposure, probable PTSD, and probable alcohol use disorder. Journal of Dual Diagnosis, 17(1), 52–63. 10.1080/15504263.2020.1854411 33308060

[bibr45-15248380241306362] Leonard S. J. McGrew S. J. Lebeaut A. Vujanovic A. A. (2023). PTSD symptom severity and alcohol use among firefighters: The role of emotion regulation difficulties. Journal of Dual Diagnosis, 19(4), 209–220. 10.1080/15504263.2023.2260324 37802496

[bibr46-15248380241306362] Lilly M. M. London M. J. (2015). Broad clinical phenotype and facets of emotion regulation in interpersonal trauma survivors. Journal of Clinical Psychology, 71(9), 885–897. 10.1002/jclp.22177 25867621

[bibr47-15248380241306362] Litz B. T. Orsillo S. M. Kaloupek D. Weathers F. (2000). Emotional processing in posttraumatic stress disorder. Journal of Abnormal Psychology, 109(1), 26–39. 10.1037//0021-843x.109.1.26 10740933

[bibr48-15248380241306362] Mahoney C. T. Cestodio V. Porter K. J. Marchant K. M. (2022). The moderating roles of emotion regulation and coping self-efficacy on the association between PTSD symptom severity and drug use among female sexual assault survivors. Psychological Trauma: Theory, Research, Practice & Policy, 36(3), 366–381. 10.1037/tra0001194 35130020

[bibr49-15248380241306362] María-Ríos C. E. Morrow J. D. (2020). Mechanisms of shared vulnerability to post-traumatic stress disorder and substance use disorders [review]. Frontiers in Behavioral Neuroscience, 14, 6. 10.3389/fnbeh.2020.00006 32082127 PMC7006033

[bibr50-15248380241306362] Marroquín B. (2011). Interpersonal emotion regulation as a mechanism of social support in depression. Clinical Psychology Review, 31(8), 1276–1290. 10.1016/j.cpr.2011.09.005 21983267

[bibr51-15248380241306362] McDermott M. J. Tull M. T. Gratz K. L. Daughters S. B. Lejuez C. W. (2009). The role of anxiety sensitivity and difficulties in emotion regulation in posttraumatic stress disorder among crack/cocaine dependent patients in residential substance abuse treatment. Journal of Anxiety Disorders, 23(5), 591–599. 10.1016/j.janxdis.2009.01.006 19233609 PMC2698460

[bibr52-15248380241306362] McGrew S. J. Raines A. M. Walker R. L. Leonard S. J. Vujanovic A. A. (2023). Posttraumatic stress, alcohol use, and alcohol use motives among non-hispanic Black/African American College students: The role of emotion regulation. Journal of Dual Diagnosis, 19(1), 3–15. 10.1080/15504263.2022.2160037 36583682 PMC10337772

[bibr53-15248380241306362] Mengin A. C. Rolling J. Porche C. Durpoix A. Lalanne L. (2022). The intertwining of posttraumatic stress symptoms, alcohol, tobacco or nicotine use, and the COVID-19 pandemic: A systematic review. International Journal of Environmental Research and Public Health, 19(21), 14546. 10.3390/ijerph192114546 PMC9658659 36361425

[bibr54-15248380241306362] NICE. (2018). Post-traumatic stress disorder. NICE Guidelines. https://www.nice.org.uk/guidance/ng116 (accessed 31 May 2024).

[bibr55-15248380241306362] Norman S. B. Haller M. Hamblen J. L. Southwick S. M. Pietrzak R. H. (2018). The burden of co-occurring alcohol use disorder and PTSD in U.S. Military veterans: Comorbidities, functioning, and suicidality. Psychology of Addictive Behaviors, 32(2), 224–229. 10.1037/adb0000348 29553778

[bibr56-15248380241306362] Office for Health Improvement & Disparities. (2023). UK clinical guidelines for alcohol treatment: Core elements of alcohol treatment. https://tinyurl.com/az8eu8xs (accessed 31 May 2024).

[bibr57-15248380241306362] Page M. J. McKenzie J. E. Bossuyt P. M. Boutron I. Hoffmann T. C. Mulrow C. D. Shamseer L. Tetzlaff J. M. Akl E. A. Brennan S. E. Chou R. Glanville J. Grimshaw J. M. Hróbjartsson A. Lalu M. M. Li T. Loder E. W. Mayo-Wilson E. McDonald S. Moher D. (2021). The PRISMA 2020 statement: an updated guideline for reporting systematic reviews. BMJ, 372, n71. 10.1136/bmj.n71 PMC8005924 33782057

[bibr58-15248380241306362] Parent M. C. Arriaga A. S. Gobble T. Wille L. (2019). Stress and substance use among sexual and gender minority individuals across the lifespan. Neurobiology of Stress, 10, 100146. 10.1016/j.ynstr.2018.100146 30937352 PMC6430403

[bibr59-15248380241306362] Patel H. Easterbrook B. D'Alessandro-Lowe A. M. Andrews K. Ritchie K. Hosseiny F. Rodrigues S. Malain A. O'Connor C. Schielke H. McCabe R. E. Nicholson A. A. Lanius R. McKinnon M. C. (2023). Associations between trauma and substance use among healthcare workers and public safety personnel during the SARS-CoV-2 (COVID-19) pandemic: The mediating roles of dissociation and emotion dysregulation. European Journal of Psychotraumatology, 14(1), 2180706. 10.1080/20008066.2023.2180706 36930578 PMC10026820

[bibr60-15248380241306362] Paulus D. J. Tran N. Gallagher M. W. Viana A. G. Bakhshaie J. Garza M. Ochoa-Perez M. Lemaire C. Zvolensky M. J. (2019). Examining the indirect effect of posttraumatic stress symptoms via emotion dysregulation on alcohol misuse among trauma-exposed Latinx in primary care. Cultural Diversity & Ethnic Minority Psychology, 25(1), 55–64. 10.1037/cdp0000226 30714767 PMC12826626

[bibr61-15248380241306362] Pebole M. M. Lyons R. C. Gobin R. L. (2022). Correlates and consequences of emotion regulation difficulties among OEF/OIF/OND veterans. Psychological Trauma: Theory, Research, Practice & Policy, 14(2), 326–335. 10.1037/tra0001046 34110894

[bibr62-15248380241306362] Petrova K. Gross J. J. (2023). The future of emotion regulation research: Broadening our field of view. Affective Science, 4(4), 609–616. 10.1007/s42761-023-00222-0 38156255 PMC10751286

[bibr63-15248380241306362] Popay J. Roberts H. Sowden A. Petticrew M. Arai L. Rodgers M. Britten N. Roen K. Duffy S. (2006). Guidance on the conduct of narrative synthesis in systematic reviews. Lancaster University.

[bibr64-15248380241306362] Radomski S. A. Read J. P. (2016). Mechanistic role of emotion regulation in the PTSD and alcohol association. Traumatology, 22(2), 113–121. 10.1037/trm0000068 27398074 PMC4933321

[bibr65-15248380241306362] Raudales A. M. Short N. A. Schmidt N. B. (2019). Emotion dysregulation mediates the relationship between trauma type and PTSD symptoms in a diverse trauma-exposed clinical sample. Personality and Individual Differences, 139, 28–33. 10.1016/j.paid.2018.10.033

[bibr66-15248380241306362] Roberts N. P. Back S. E. Mueser K. T. Murray L. K. (2020). Treatment considerations for PTSD comorbidities. In Forbes D. Bisson J. I. Monson C. M. Berliner L. (Eds.), Effective treatments for PTSD: Practice guidelines from the International Society for Traumatic Stress Studies. The Guilford Press.

[bibr67-15248380241306362] Roberts N. P. Lotzin A. Schäfer I. (2022). A systematic review and meta-analysis of psychological interventions for comorbid post-traumatic stress disorder and substance use disorder. European Journal of Psychotraumatology, 13(1), 2041831. 10.1080/20008198.2022.2041831 PMC9090345 35558682

[bibr68-15248380241306362] Roberts N. P. Roberts P. A. Jones N. Bisson J. I. (2016). Psychological therapies for post-traumatic stress disorder and comorbid substance use disorder. Cochrane Database of Systematic Reviews, 4, CD010204. 10.1002/14651858.CD010204.pub2 PMC8782594 27040448

[bibr69-15248380241306362] Sanchis-Sanchis A. Grau M. D. Moliner A.-R. Morales-Murillo C. P. (2020). Effects of age and gender in emotion regulation of children and adolescents. Frontiers in Psychology, 11, 946. 10.3389/fpsyg.2020.00946 32528367 PMC7265134

[bibr70-15248380241306362] Schäfer I. Najavits L. M. (2007). Clinical challenges in the treatment of patients with posttraumatic stress disorder and substance abuse. Current Opinion in Psychiatry, 20(6), 614–618. 10.1097/YCO.0b013e3282f0ffd9 17921765

[bibr71-15248380241306362] Seligowski A. V. Lee D. J. Bardeen J. R. Orcutt H. K. (2015). Emotion regulation and posttraumatic stress symptoms: A meta-analysis. Cognitive Behaviour Therapy, 44(2), 87–102. 10.1080/16506073.2014.980753 25421727

[bibr72-15248380241306362] Shuman V . (2013). Studying the social dimension of emotion regulation.Frontiers in Psychology, 4, 922. 10.3389/fpsyg.2013.00922 PMC3851788 24367350

[bibr73-15248380241306362] Silva M. Loureiro A. Cardoso G. (2016). Social determinants of mental health: A review of the evidence. European Journal of Psychiatry, 30, 259–292.

[bibr74-15248380241306362] Sloan E. Hall K. Moulding R. Bryce S. Mildred H. Staiger P. K. (2017). Emotion regulation as a transdiagnostic treatment construct across anxiety, depression, substance, eating and borderline personality disorders: A systematic review. Clinical Psychology Review, 57, 141–163. 10.1016/j.cpr.2017.09.002 28941927

[bibr75-15248380241306362] Stellern J. Xiao K. B. Grennell E. Sanches M. Gowin J. L. Sloan M. E. (2023). Emotion regulation in substance use disorders: A systematic review and meta-analysis. Addiction, 118(1), 30–47. 10.1111/add.16001 35851975 PMC10087816

[bibr76-15248380241306362] Stern C. Lizarondo L. Carrier J. Godfrey C. Rieger K. Salmond S. Apóstolo J. Kirkpatrick P. Loveday H. (2020). Methodological guidance for the conduct of mixed methods systematic reviews. JBI Evidence Synthesis, 18(10). 10.11124/JBISRIR-D-19-00169 10.11124/JBISRIR-D-19-00169 32813460

[bibr77-15248380241306362] Tripp J. C. McDevitt-Murphy M. E. (2015). Emotion dysregulation facets as mediators of the relationship between PTSD and alcohol misuse. Addictive Behaviors, 47, 55–60. 10.1016/j.addbeh.2015.03.013 25864136 PMC4420630

[bibr78-15248380241306362] Tull M. T. Aldao A . (2015). Editorial overview: New directions in the science of emotion regulation. Current Opinion in Psychology, 3. 10.1016/j.copsyc.2015.03.009

[bibr79-15248380241306362] Tull M. T. Bardeen J. R. DiLillo D. Messman-Moore T. Gratz K. L. (2015). A prospective investigation of emotion dysregulation as a moderator of the relation between posttraumatic stress symptoms and substance use severity. Journal of Anxiety Disorders, 29, 52–60. 10.1016/j.janxdis.2014.11.003 25483275 PMC4749400

[bibr80-15248380241306362] Wang Y. Chung M. C. Wang N. Yu X. Kenardy J. (2021). Social support and posttraumatic stress disorder: A meta-analysis of longitudinal studies. Clinical Psychology Review, 85, 101998. 10.1016/j.cpr.2021.101998 33714168

[bibr81-15248380241306362] Warner N. Murphy M. (2022). Dialectical behaviour therapy skills training for individuals with substance use disorder: A systematic review. Drug and Alcohol Review, 41(2), 501–516. 10.1111/dar.13362 34337811

[bibr82-15248380241306362] Wegen K. S. van Dijke A. Aalbers A. Zedlitz A. (2017). Dissociation and under-regulation of affect in patients with posttraumatic stress disorder with and without a co-morbid substance use disorder. European Journal of Trauma & Dissociation, 1(4), 227–234. 10.1016/j.ejtd.2017.06.001

[bibr83-15248380241306362] Weiss N. H. Brick L. A. Schick M. R. Forkus S. R. Raudales A. M. Contractor A. A. Sullivan T. P. (2022). Posttraumatic stress disorder strengthens the momentary associations between emotion dysregulation and substance use: A micro-longitudinal study of community women experiencing intimate partner violence. Addiction, 117(12), 3150–3169. 10.1111/add.15992 35792057 PMC12090078

[bibr84-15248380241306362] Weiss N. H. Darosh A. G. Contractor A. A. Forkus S. R. Dixon-Gordon K. L. Sullivan T. P. (2018). Heterogeneity in emotion regulation difficulties among women victims of domestic violence: A latent profile analysis. Journal of Affective Disorders, 239, 192–200. 10.1016/j.jad.2018.07.009 30014959 PMC12038377

[bibr85-15248380241306362] Weiss N. H. Forkus S. R. Raudales A. M. Schick M. R. Contractor A. A. (2020). Alcohol misuse to down-regulate positive emotions: A cross-sectional multiple mediator analysis among US military veterans. Addictive Behaviors, 105. 10.1016/j.addbeh.2020.106322 PMC7059215 32006684

[bibr86-15248380241306362] Weiss N. H. Goncharenko S. Raudales A. M. Schick M. R. Contractor A. A. (2021). Alcohol to down-regulate negative and positive emotions: Extending our understanding of the functional role of alcohol in relation to posttraumatic stress disorder. Addictive Behaviors, 115. 10.1016/j.addbeh.2020.106777 PMC7855539 33359633

[bibr87-15248380241306362] Weiss N. H. Kiefer R. Goncharenko S. Raudales A. M. Forkus S. R. Schick M. R. Contractor A. A. (2021). Emotion regulation and substance use: A meta-analysis. Drug and Alcohol Dependence, 109131. 10.1016/j.drugalcdep.2021.109131 PMC8714680 34864568

[bibr88-15248380241306362] Weiss N. H. Schick M. R. Contractor A. A. Dixon-Gordon K. L. (2019). Posttraumatic stress disorder and substance use: Identifying the underlying role of difficulties regulating positive emotions. Addictive Behaviors, 96, 119–126. 10.1016/j.addbeh.2019.04.029 31075729 PMC13103676

[bibr89-15248380241306362] Weiss N. H. Thomas E. D. Schick M. R. Reyes M. E. Contractor A. A. (2022). Racial and ethnic differences in emotion regulation: A systematic review. Journal of Clinical Psychology, 78(5), 785–808. 10.1002/jclp.23284 34841522 PMC9035029

[bibr90-15248380241306362] Weiss N. H. Tull M. T. Anestis M. D. Gratz K. L. Weiss N. H. Tull M. T. Anestis M. D. Gratz K. L. (2013). The relative and unique contributions of emotion dysregulation and impulsivity to posttraumatic stress disorder among substance dependent inpatients. Drug & Alcohol Dependence, 128(1/2), 45–51. 10.1016/j.drugalcdep.2012.07.017 22917752 PMC3513512

[bibr91-15248380241306362] Weiss N. H. Tull M. T. Lavender J. Gratz K. L. (2013). Role of emotion dysregulation in the relationship between childhood abuse and probable PTSD in a sample of substance abusers. Child Abuse & Neglect, 37(11), 944–954. 10.1016/j.chiabu.2013.03.014 23643388

[bibr92-15248380241306362] Wen A. Rao U. Kinney K. L. Yoon K. L. Morris M. (2024). Diversity in emotion regulation strategy use: Resilience against posttraumatic stress disorder. Behaviour Research and Therapy, 172, 104441. 10.1016/j.brat.2023.104441 38091721 PMC11292606

[bibr93-15248380241306362] Westphal M. Aldao A. Jackson C. (2017). Emotion dysregulation in comorbid posttraumatic stress disorder and substance use disorders: A narrative review. Military Psychology, 29(3), 216–233. 10.1037/mil0000157

[bibr94-15248380241306362] Witte T. H. Weymouth B. B. Gajos J. M. Penunuri A. Levy S. (2020). Trauma exposure and problem drinking in late adolescence: A latent profile analysis. Journal of Traumatic Stress, 33(6), 1048–1059. 10.1002/jts.22599 33038904

[bibr95-15248380241306362] Wolff S. Holl J. Stopsack M. Arens E. A. Höcker A. Staben K. A. Hiller P. Klein M. Schäfer I. Barnow S. (2016). Does emotion dysregulation mediate the relationship between early maltreatment and later substance dependence? Findings of the CANSAS study. European Addiction Research, 22(6), 292–300. 10.1159/000447397 27438781

[bibr96-15248380241306362] Wolitzky-Taylor K. Smit T. Vujanovic A. A. Zvolensky M. J. (2023). Transdiagnostic processes linking posttraumatic stress disorder symptoms to alcohol use severity. Journal of Dual Diagnosis, 19(2–3), 97–110. 10.1080/15504263.2023.2225373 37389859

