# Supplementary material for: Emotion Regulation in the Association Between Posttraumatic Stress Disorder and Substance Use: A Systematic Review With Narrative Synthesis
Source: Trauma Violence Abuse. 2024 Dec 30;27(1):3–21. doi: 10.1177/15248380241306362 (PMC12662837; doi:10.1177/15248380241306362)
Supplement: sj-docx-1-tva-10.1177_15248380241306362 – Supplemental material for Emotion Regulation in the Association Between Posttraumatic Stress Disorder and Substance Use: A Systematic Review With Narrative Synthesis [file sj-docx-1-tva-10.1177_15248380241306362.docx]

#### **Supplementary Appendix A: Full list of search terms used in systematic review.**

| **1. Emotional regulation** | | |
| --- | --- | --- |
| 1a. Subject Headings/ Thesaurus Terms | PsycInfo | Emotional Regulation |
|  | Medline | Emotional Regulation |
|  | Embase | Emotional Regulation |
|  | CINAHL | Emotional Regulation |
|  | ASSIA | Emotional Regulation |
|  | Web of Science | N/A |
| 1b. Key words for Medline, Embase and PsycInfo | “emotion* adj3 regulat*” OR “emotion* adj3 dysfunction” OR “emotion* adj3 dysregulat*” OR “affect* adj3 regulat*” OR “affect* adj3 dysfunction” OR “affect*adj3 dysregulat*” OR “difficult* adj3 regulat*” | |
| 1c. Key words for Web of Science and ASSIA | “emotion* NEAR/3 regulat*” OR “emotion* NEAR/3 dysfunction” OR “emotion* NEAR/3 dysregulat*” OR “affect* NEAR/3 regulat*” OR “affect* NEAR/3 dysfunction” OR “affect* NEAR/3 dysregulat*” OR “difficult* NEAR/3 regulat*” | |
| 1d. Key words for CINAHL | “emotion* N3 regulat*” OR “emotion* N3 dysfunction” OR “emotion* N3 dysregulat*” OR “affect* N3 regulat*” OR “affect* N3 dysfunction” OR “affect* N3 dysregulat*” OR “difficult* N3 regulat*” | |
| **2. PTSD** | | |
| 2a. Subject Headings/ Thesaurus Terms | PsycInfo | Posttraumatic Stress Disorder |
|  | Medline | Stress Disorders, Post-Traumatic |
|  | Embase | posttraumatic stress disorder |
|  | CINAHL | Stress Disorders, Post-Traumatic |
|  | ASSIA | posttraumatic stress disorder |
|  | Web of Science | N/A |
| 2b. Key words | “posttraumatic stress” OR “post-traumatic stress” OR PTSD OR “post traumatic stress” OR “posttraumatic stress disorder” | |
| **3. Substance use** |  |  |
| 3a. Subject Headings/ Thesaurus Terms | PsycInfo | Substance Use Disorder  Drug Usage  Alcohol Abuse |
|  | Medline | Substance-Related Disorders Drug Misuse  Alcohol Drinking  Illicit Drugs |
|  | Embase | Drug dependence  Substance use Drug use Substance abuse  Drug abuse  Illicit Drug |
|  | CINAHL | Substance Use Disorders  Alcohol Drinking  Street Drugs |
|  | ASSIA | Drug dependency Substance Abuse  Substance abuse disorders  Alcohol consumption |
|  | Web of Science | N/A |
| 3b. Key words for Medline, Embase and PsycInfo | addict* OR drug* OR alcohol* OR “hazardous drink*” OR AUD OR hallucinogen* OR narcotic* OR stimulant* OR psychotropic* OR psychedelic* OR “substance adj2 use*” OR “substance adj2 misuse*” OR “substance adj2 abuse*” OR SUD OR “substance use disorder” OR “substance adj2 dependen*” OR cannabis OR marijuana OR “hash*” OR “cocaine” OR “crack” OR “heroin” OR “opioid*” OR “opiate*” OR “OUD” OR “amphetamine*” OR “ecstasy” OR “MDMA” or “LSD” or “methamphetamine*” OR “new psychoactive substance” OR “NPS” OR “benzodiazepine*” OR “inhalant*” OR “prescription drug*” OR “polydrug” OR “polysubstance” OR “psychoactive*” OR “barbiturate*” OR “ketamine” OR “mephedrone” | |
| 3c. Key words for Web of Science and ASSIA | “addict*” OR “drug*” OR “alcohol*” OR “hazardous drink*” OR “AUD” OR “hallucinogen*” OR “narcotic*” OR “stimulant*” OR “psychotropic*” OR “psychedelic*” OR “substance NEAR/2 use*” OR “substance NEAR/2 misuse*” OR “substance NEAR/2 abuse*” OR “SUD” OR “substance use disorder” OR “substance NEAR/2 dependen*” OR “cannabis” OR “marijuana” OR “hash*” OR “cocaine” OR “crack” OR “heroin” OR “opioid*” OR “opiate*” OR “OUD” OR “amphetamine*” OR “ecstasy” OR “MDMA” or “LSD” or “methamphetamine*” OR “new psychoactive substance” OR “NPS” OR “benzodiazepine*” OR “inhalant*” OR “prescription drug*” OR “polydrug” OR “polysubstance” OR “psychoactive*” OR “barbiturate*” OR “ketamine” OR “mephedrone” | |
| 3d. Key words for CINAHL | “addict*” OR “drug*” OR “alcohol*” OR “hazardous drink*” OR “AUD” OR “hallucinogen*” OR “narcotic*” OR “stimulant*” OR “psychotropic*” OR “psychedelic*” OR “substance N2 use*” OR “substance N2 misuse*” OR “substance N2 abuse*” OR “SUD” OR “substance use disorder” OR “substance N2 dependen*” OR “cannabis” OR “marijuana” OR “hash*” OR “cocaine” OR “crack” OR “heroin” OR “opioid*” OR “opiate*” OR “OUD” OR “amphetamine*” OR “ecstasy” OR “MDMA” or “LSD” or “methamphetamine*” OR “new psychoactive substance” OR “NPS” OR “benzodiazepine*” OR “inhalant*” OR “prescription drug*” OR “polydrug” OR “polysubstance” OR “psychoactive*” OR “barbiturate*” OR “ketamine” OR “mephedrone” | |
