# Supplementary material for: Emotion Regulation in the Association Between Posttraumatic Stress Disorder and Substance Use: A Systematic Review With Narrative Synthesis
Source: Trauma Violence Abuse. 2024 Dec 30;27(1):3–21. doi: 10.1177/15248380241306362 (PMC12662837; doi:10.1177/15248380241306362)
Supplement: sj-docx-2-tva-10.1177_15248380241306362 – Supplemental material for Emotion Regulation in the Association Between Posttraumatic Stress Disorder and Substance Use: A Systematic Review With Narrative Synthesis [file sj-docx-2-tva-10.1177_15248380241306362.docx]

**Supplementary Appendix C: Measures of emotional regulation, PTSD, substance use, and trauma administered in the included studies.**

| **Author (Year)** | **Measures** | | | |
| --- | --- | --- | --- | --- |
|  | **Emotional Regulation** | **PTSD** | **Substance Use** | **Trauma** |
| Aase (2018) | Emotion Regulation Questionnaire | PTSD Checklist - Military Version | Alcohol Use Disorders Identification Test | Combat Exposure Scale |
| Bornavalova (2009) | Difficulties in Emotion Regulation Scale* | PTSD Checklist - Civilian Version | Drug Use Questionnaire | The PTSD Checklist - Civilian Version |
| Christ (2022) | Emotion Regulation Questionnaire | PTSD Checklist for DSM-5 | Alcohol Use Disorders Identification Test | The Stressful Life Events Questionnaire The PTSD checklist for DSM-5 |
| Fairholme (2013) | Difficulties in Emotion Regulation Scale** | PTSD Checklist Impact of Events Scale-Revised | Alcohol Dependence Scale | Self-reported trauma |
| Feingold (2021) | Emotion Regulation Questionnaire | PTSD Checklist for DSM-5 | Alcohol Use Disorders Identification Test | Combats Experience Scale |
| Goldstein (2017) | Difficulties in Emotion Regulation Scale | Clinician Administered PTSD Scale | MINI International Neuropsychiatric Interview - current alcohol dependence | Traumatic Events Inventory |
| Gonacharenko (2019) | Difficulties in Emotion Regulation Scale Difficulties in Emotion Regulation Scale - Positive | PTSD Checklist for DSM-5 Primary Care PTSD Screen for DSM-5 | Alcohol Use Disorders Identification Test - Consumption | Life Events Checklist for DSM-5 |
| Hien (2017) | Difficulties in Emotion Regulation Scale | Clinician Administered PTSD Scale Modified PTSD Symptom Scale - Self Report | The Structured Clinical Interview for DSM-IV for Axis I disorders Substance Use Inventory | Clinician Administered PTSD Scale |
| Holzhauer (2017) | Difficulties in Emotion Regulation Scale | Psychiatric Research Interview for Substance and Mental Disorders Modified PTSD Symptom Scale | Psychiatric Research Interview for Substance and Mental Disorders  Timeline Follow-back Interview to calculate % days abstinent | Not reported |
| Klanecky (2016) | Difficulties in Emotion Regulation Scale | PTSD Checklist - Civilian Version | Alcohol Use Disorders Identification Test | Early Trauma Inventory Self-Report Short Form - sexual trauma items |
| Klemanski (2012) | Difficulties in Emotion Regulation Scale | Clinician Administered PTSD Scale | Michigan Alcohol Screening Test | Combat Exposure Scale Childhood Trauma Questionnaire Traumatic Life Events Questionnaire |
| Lebeaut (2021) | Difficulties in Emotion Regulation Scale | PTSD Checklist for DSM-5 | Alcohol Use Disorders Identification Test | Life Events Checklist for DSM-5 |
| Leonard (2023) | Brief Difficulties in Emotion Regulation Scale | PTSD Checklist for DSM-5 | Alcohol Use Disorders Identification Test | Life Events Checklist for DSM-5 |
| Lilly (2015) | Difficulties in Emotion Regulation Scale | Posttraumatic Stress Diagnostic Scale | Michigan Alcohol Screening Test | Traumatic Life Events Questionnaire - some items |
| Mahoney (2022) | Difficulties in Emotion Regulation Scale | PTSD Checklist for DSM-5 | Drug Use Disorders Identification Test | Sexual Experiences Survey-Short Form |
| McDermott (2009) | Difficulties in Emotion Regulation Scale | PTSD Checklist | Self-report measure of substance use modelled on Alcohol Use Disorders Identification Test | Life Events Checklist |
| McGrew (2022) | Brief Difficulties in Emotion Regulation Scale | PTSD Checklist for DSM-5 | Alcohol Use Disorders Identification Test | Life Events Checklist for DSM-5 |
| Patel (2023) | Difficulties in Emotion Regulation Scale | PTSD Checklist for DSM-5 | Alcohol Use Disorders Identification Test Drug Use Disorders Identification Test | PTSD Checklist for DSM-5 |
| Paulus (2019) | Difficulties in Emotion Regulation Scale** | Post Traumatic Diagnostic Scale Inventory of Anxiety and Depression Symptoms - trauma subscale Mini International Neuropsychiatric Interview | Alcohol Use Disorders Identification Test Mini International Neuropsychiatric Interview | Post Traumatic Diagnostic Scale |
| Pebole (2022) | Difficulties in Emotion Regulation Scale | PTSD Checklist - Specific | Alcohol Use Disorders Identification Test | Life Events Checklist for DSM-5 |
| Radomski (2016) | Difficulties in Emotion Regulation Scale | Clinician Administered PTSD Scale | Self-report based on Daily Drinking Questionnaire. | Life Events Checklist |
| Tripp (2015) | Difficulties in Emotion Regulation Scale | PTSD Checklist - Military Version | Alcohol Use Disorders Identification Test | PTSD Checklist - Military |
| Tull (2015) | Difficulties in Emotion Regulation Scale Modified version of the Paced Auditory Serial Addition Task - Computerised | PTSD Checklist - Civilian Version | Drug Use Questionnaire | Life Events Checklist |
| Wegen (2017) | Dutch self-report version of the Structured Interview for Disorders of Extreme Stress Not Otherwise Specified Revised - Affect Dysregulation Subscale | Dutch version of the Self-Rating Inventory for Posttraumatic Stress Disorder  Clinical interview following the DSM-IV criteria for PTSD, symptomatology as well as functional impairment. | Dutch version of the Europe Addiction Severity Index Additional explorative question asking about subjective reason for substance in relation to influencing emotions. | Traumatic Experiences Checklist - Dutch |
| Weiss (2022a) | Difficulties in Emotion Regulation Scale*** Difficulties in Emotion Regulation Scale - Positive *** | Structured Clinical Interview for DSM-5 Disorders | Self-report alcohol and drug use completed 3 x a day for 30 days | Self-reported trauma |
| Weiss (2018) | Difficulties in Emotion Regulation Scale**** Difficulties in Emotion Regulation Scale - Positive | Post Traumatic Diagnostic Scale | Alcohol Use Disorders Identification Test Drug Abuse Screening Test | Revised Conflict Tactics Scale - 12 items Psychological Maltreatment of Women Scale - 14 items Sexual Experiences Survey |
| Weiss (2020) | Emotional Avoidance Questionnaire - Positive | PTSD Checklist for DSM-5 | Alcohol Use Disorders Identification Test | Life Events Checklist for DSM-5 |
| Weiss (2021a) | The Revised Regulatory Emotional Self-Efficacy Scale | PTSD Checklist for DSM-5 | Alcohol Use Disorders Identification Test | Life Events Checklist for DSM-5 |
| Weiss (2019) | Difficulties in Emotion Regulation Scale - Positive | PTSD Checklist for DSM-5 | Alcohol Use Disorders Identification Test - Consumption Drug Abuse Screening Test | Life Events Checklist for DSM-5 |
| Weiss (2013a) | Difficulties in Emotion Regulation Scale | Clinician Administered PTSD Scale | Structured Clinical Interview for DSM-IV Axis I disorders | Clinician Administered PTSD Scale |
| Weiss (2013b) | Difficulties in Emotion Regulation Scale | The PTSD Checklist-Civilian Version | Not measured - substance use disorder patients | Childhood Trauma Questionnaire - Short Form |
| Witte (2020) | Difficulties in Emotion Regulation Scale | Post Traumatic Diagnostic Scale | Daily Drinking Questionnaire The CAGE Questionnaire  Modified version of the first item on the Alcohol Use Disorder Identification Test | Post Traumatic Diagnostic Scale |
| Wolitzky-Taylor (2023) | Brief Difficulties in Emotion Regulation Scale | PTSD Checklist for DSM-5 | Alcohol Use Disorders Identification Test | Life Events Checklist for DSM-5 |

| * Awareness, impulse and clarity subscales only | |
| --- | --- |
| ** Excluding Awareness subscale | |
| *** Abbreviated 6 item momentary versions | |
| **** Acceptance, goals and impulse subscales only |  |
