# Supplementary material for: Emotion Regulation in the Association Between Posttraumatic Stress Disorder and Substance Use: A Systematic Review With Narrative Synthesis
Source: Trauma Violence Abuse. 2024 Dec 30;27(1):3–21. doi: 10.1177/15248380241306362 (PMC12662837; doi:10.1177/15248380241306362)
Supplement: sj-docx-3-tva-10.1177_15248380241306362 – Supplemental material for Emotion Regulation in the Association Between Posttraumatic Stress Disorder and Substance Use: A Systematic Review With Narrative Synthesis [file sj-docx-3-tva-10.1177_15248380241306362.docx]

**Supplementary Appendix D. Key findings from the included studies on gender, trauma type and social factors.**

| Key finding | Studies |
| --- | --- |
| Gender | |
| Gender or sex at birth included as a covariate | Christ (2022); Leonard (2023); Paulus (2019); Patel (2023); Radomski (2016); Wegen (2017); Weiss (2013a); Weiss (2013b); Weiss (2019); Weiss (2021a); Witte (2020); Wolitzsky-Taylor (2023) |
| Differences in emotional regulation observed when comparing males to full mixed-gender sample | Tripp (2015); Weiss (2013b) |
| Gender differences in emotional regulation reported | Bornavalova (2009); Goncharenko (2019) |
| Trauma exposure | |
| Trauma type included as a covariate | Aase (2018); Feingold 2021); Goldstein (2017); Klemanski (2012); Leonard (2023); Mahoney (2022); McGrew (2022); Patel (2023); Paulus (2019); Weiss (2021a) |
| No differences in trauma type between study groups | McDermott (2009); |
| Differences in trauma type observed between study groups | Klemanski (2012); Lilly (2015); Wegen (2017); Weiss (2013b); Witte (2020) |
| Childhood and adolescent trauma was more strongly related to emotional regulation difficulties than cumulative trauma | Klanecky (2016) |
| Trauma type not related to PTSD symptoms, emotional regulation, or alcohol use. | Pebole (2022) |
| Social factors | |
| Social factors included as covariates | Feingold (2021); Klemanski (2012); Lebeaut (2021); Leonard (2023); Patel (2023); Paulus (2019); Weiss (2022a) |
| No differences observed between groups | Lilly (2015); McDermott (2009), Wegen (2017); Weiss (2013a); Weiss (2013b) |
| Differences observed between study groups | Klemanski (2012); Lebeaut (2021) |
| Social factors not related to PTSD, substance use or emotional regulation | Bornavalova (2009) |
| Relationships related to emotional regulation | Holzhauer (2017); Lebeaut (2021) |
| Occupational stress and duration significantly related to PTSD, substance use or emotional regulation | Leonard (2023) |
