# Supplementary material for: Emotion Regulation in the Association Between Posttraumatic Stress Disorder and Substance Use: A Systematic Review With Narrative Synthesis
Source: Trauma Violence Abuse. 2024 Dec 30;27(1):3–21. doi: 10.1177/15248380241306362 (PMC12662837; doi:10.1177/15248380241306362)
Supplement: sj-docx-4-tva-10.1177_15248380241306362 – Supplemental material for Emotion Regulation in the Association Between Posttraumatic Stress Disorder and Substance Use: A Systematic Review With Narrative Synthesis [file sj-docx-4-tva-10.1177_15248380241306362.docx]

#### **Supplementary Appendix B: Sample and demographic characteristics of the included studies.**

| **Author (Year), Country, Study Design** |  | |  | | **Sample Characteristics** | | | | **Trauma type** | **Substance (s)** |
| --- | --- | --- | --- | --- | --- | --- | --- | --- | --- | --- |
|  | **Population** | **Comparison groups** | | **Total N** | | **Mean Age (SD)** | **Sex/Gender*** | **Ethnicity %** |  |  |
| Aase (2018), USA, Prospective longitudinal | Military veterans | NA | | 71 | | 33.13 (6.55) | 83.1% male | Caucasian 26.8%, African American 32.4%, Hispanic 36.6%, Other or biracial 0.2% | Military | Alcohol |
| Bornavalova (2009), USA, Cross-sectional | Clinical - Alcohol & drug treatment inpatients | NA | | 182 | | Men 43.43 (10.68)  Women 41.94 (7.64) | 72% male | 91.4% African American | Not reported | Alcohol and Drugs |
| Christ (2022), USA, Cross-sectional | Community - trauma exposed adults | NA | | 334 | | 36.05 (12.71) | 63.5% male, 36.2% female | White 77.5%, non-Hispanic 80.2% | Various | Alcohol |
| Fairholme (2013), USA, Cross-sectional | Clinical - in residential substance use treatment & met cut-offs for probable PTSD and problematic alcohol use | NA | | 220 | | 33.7 (10.2) | 47.7% female | White 77.7%, Black/African American 20.0%, Other 1.4% | Not reported | Alcohol |
| Feingold (2021), Israel, Cross-sectional | Military veterans | NA | | 189 | | 30.03 (4.16) | 100% male | Not reported | Military | Alcohol |
| Goldstein (2017), USA, Cross-sectional | Community - trauma exposed adults | NA | | 260 | | 39.76 (11.73) | 100% female | African American 96.9%, Caucasian 1.1%, Mixed/Other 2% | Not reported | Alcohol |
| Gonacharenko (2019), USA, Cross-sectional | Community - trauma exposed adults (Amazon M-Turk) | NA | | 475 | | 35.62 (11.07) | 43.2% male, 55.4% female, 0.8% female to male transgender, 0.1% | Ethnicity Hispanic/Latinx 12.8%, Not Hispanic/Latinx 85.5%  Race White 77.0%, African American/Black 9.1%, Asian 10.9%, American Indian/Alaskan Native 4.4% | Various | Alcohol |
| Hien (2017), USA, Secondary analysis - RCT | Clinical - community substance use services - co-occurring SUD and full or subthreshold PTSD | Receiving Concurrent Treatment with Prolonged Exposure (COPE)   Receiving Relapse Prevention Therapy (RPT)  Active Monitoring Control Group (AMCG) | | Total: 110  COPE: 39  RPT: 43  AMCG:28 | | COPE: 43.08 (10)  RPT: 44.21 (9.05)  AMCG: 47.18 (8.21) | COPE: 28.2% female  RPT: 37.2% female  AMCG: 46.4% female | COPE Black/African American 53.8%, Hispanic/Latino 25.6%, White 15.4%, Other 5.1%  RPT Black/African American 65.1%, Hispanic/Latino 29.9%, White 14%, Other 5.1%  AMCG Black/African American 57.1%, Hispanic/Latino 10.7%, White 28.6%, Other 3.6% | Various | Alcohol and Drugs |
| Holzhauer (2017), Secondary analysis - RCT | Clinical - community substance use services - co-occurring alcohol dependence and major depression | NA | | 48 | | 37.4 (11.5) | 100% women | White/Caucasian 62.5%, Black/African American 31.3%, Multiple racial identities 6.3% | Not reported | Alcohol |
| Klanecky (2016), USA, Cross-sectional | College students - had violated 'dry campus' policy | NA | | 213 | | 19.56 (1.12) | 63.4% men, 36.6% women | European African 84% | Childhood & Adolescent sexual abuse | Alcohol |
| Klemanski (2012), USA, Cross-sectional | Military Active Duty | PTSD (combat exposed and met PTSD criteria)  Combat resilient (combat exposed but didn't meet PTSD criteria)   Pre-deployment:(not combat exposed) | | Total: 44   PTSD group: 14  Resilient group: 15  Pre-deployment group: 15 | | PTSD group: 24.29 (2.55)  Combat resilient: 24.20 (2.70)  Pre-deployment: 25.47 (5.85) | 100% male | PTSD group Caucasian 57.1%, African American 7.1%, Hispanic/Latino 28.6%, Asian American 7.1%  Resilient group Caucasian 53.3%, African American 6.7%, Hispanic/Latino 13.3%, Asian American 13.3%, Other 13.3%  Pre-deployment Caucasian 53.3%, African American 26.7%, Hispanic/Latino 13.3%, Asian American 0.0%, Other 6.7% | Various | Alcohol |
| Lebeaut (2021), USA, Cross-sectional | Firefighters | Trauma exposed   PTSD only (probable PTSD)  AUD only (probable AUD)  PTSD-AUD (probable PTSD and AUD | | Total: 657  Probable PTSD-AUD: 27  Probably PTSD alone: 35  Probable AUD alone: 125  Trauma exposure only: 470 | | Trauma exposed 39.04 (8.77)  PTSD only 38.6 (8.27)  AUD only 37.81 (8.05)  PTSD-AUD 37.59 (8.07) | Trauma exposed 93.8% female, 6.2% male  PTSD only 100% male  AUD only 4.8% female, 95.2% male  PTSD-AUD 14.8% female, 85.2% male | Trauma exposed  White 77%, Hispanic Latino 25.5%, Black African American 11.5%, Other 6.6%, American Indian/Alaskan Native 2.3%, Asian 1.7%, Native Hawaiian/Pacific Islander 0.2%  PTSD only White 71.4%, Hispanic Latino 28.6%. Black African American 14.3%, Other 11.4%, American Indian/Alaskan Native 0% , Asian 2.9%, Native, Hawaiian/Pacific Islander 0%  AUD only  White 82.4%, Hispanic Latino 27.2%, Black African American 6.4%, Other 9.6%, American Indian/Alaskan Native 0%, Asian 1.6%, Native Hawaiian/Pacific Islander 0%  PTSD-AUD White 77.8%, Hispanic Latino 25.9%, Black African American 11.1%, Other 7.4%, American Indian/Alaskan Native 3.7%, Asian 0%, Native Hawaiian/Pacific Islander 0% | Not reported | Alcohol |
| Leonard (2023), USA, Cross-sectional | Firefighters | N/A | | 685 | | 38.7 (8.6) | 93.6% male, 5.7% female, 0.7% transgender | Ethnicity Hispanic/Latino 26.0%, Non-Hispanic/Latino 74.0%  Race White 77.7%, Black/African American 11.1%, Other 7.7%, American Indian/Alaskan Native 1.8%, Asian 1.6%, Native Hawaiian/Pacific Islander 0.1%. | Various | Alcohol |
| Lilly (2015), USA, Cross-sectional | Community - Interpersonal trauma survivors | NA | | 205 | | 31.53 (10.19) | 100% female | European American 44.4%, African American 41.5%, Native American 2%, Asian /Asian American 1.5%, Hispanic, American 4.4%, Biracial 4.9% other 1.5% | Interpersonal trauma | Alcohol |
| Mahoney (2022), USA, Cross-sectional | College students who reported at least one incident of attempted or completed rape | NA | | 287 | | 23.14 (7.08) | 100% women | Caucasian 83.6%, Latina/Hispanic 17.4%, American Indian/Alaska Native 2.1% | Sexual abuse | Drugs |
| McDermott (2009), USA, Cross-sectional | Clinical - crack cocaine dependent inpatients in residential treatment | Crack cocaine dependent and PTSD  Crack cocaine dependent without -PTSD | | Total: 58  Probable PTSD: 25  non-PTSD:33 | | 45.43 (7.04) | 70.7% male | Black/African American 89.7%, White 8.6%, Other 1.7% | Various | Drugs (crack/cocaine) |
| McGrew (2022), USA, Cross-sectional | College students | N/A | | 282 | | 22.36 (4.71) | 77.5% female, 21.5% male | Black/African American 100% | Various | Alcohol |
| Patel (2023), Canada, Cross-sectional | Healthcare workers and public safety personnel | Healthcare workers (HCWs)  Public Safety Personnel (PSPs) | | Total: 498  HCWs: 299  PSPs: 199 | | Not reported | HCWs: 91.6% female  PSPs: 39.7% female | Race  HCWs African/Caribbean 0.02%, Caucasian 86.6%, East Asian 0.03%, Indigenous 0.02%, Latin American 0%, Pacific Islander 0%, South East Asian 0.02%, Other 0.05%  PSPs African/Caribbean 0%, Caucasian 94.4%, East Asian 0.005%, Indigenous 0.03%, Latin American 0.01%, Pacific Islander 0%, South East Asian 0.01%, Other 0.01% | Not reported | Alcohol and Drugs |
| Paulus (2019), USA, Cross-sectional | Community -trauma exposed adults | NA | | 238 | | 38.04 (11.06) | 88.7% female | Mexican/Mexican American 52.9%, Central American 30.3%, South American 5.9%, American/born in America 5%, Cuban 2.5%, Other 2.9% | Various | Alcohol |
| Pebole (2022), USA, Cross-sectional | Military veterans | NA | | 74 | | 40.9 (12.97) | 93.2% male, 5.5.% female, 1.4% missing | Ethnicity: Hispanic/Latino 11.0%, Non-Hispanic/Latino 84.9, Unanswered/Missing 5.4%  Race: American/Alaska Native 4.1%, Asian 5.5%, Black/African American 26.0%, Native Hawaiian/Pacific Islander 4.1%, White 46.6%, Biracial 1.4%, Other 1.4, Unanswered/Missing 12.1% | Not reported | Alcohol |
| Radomski (2016), USA, Cross-sectional | College students | No trauma  Trauma exposure, no PTSD  PTSD | | Total: 466  No trauma: 182  Trauma exposure, no PTSD: 171  PTSD: 113 | | 19.5 (1.42) | 53% female | Caucasian 72% | Various | Alcohol |
| Tripp (2015), USA, Cross-sectional | Military veterans | NA | | 139 | | 35.04 (9.96) | 89% male | African American 46%, Caucasian 46%, multi-ethnic 4%, Asian 1%, Hispanic (1%, Native American 1%, not specified 1% | Military | Alcohol |
| Tull (2015), USA, Prospective longitudinal | Community - trauma exposed adults | NA | | 106 | | 21.9 (2) | 100% women | African American 76.7%, White 21.4%, Multiracial 2.8%, Latina 1.9% | Various | Alcohol and drugs |
| Wegen (2017), The Netherlands, Cross-sectional | Clinical - attending in or outpatient drug and alcohol treatment | PTSD only   SUD only   PTSD-SUD | | Total: 243  PTSD only: 103  SUD only:58  PTSD-SUD: 82 | | Total 37.11 (32.30)  PTSD 32.30 (9.33)  SUD 41.93 (9.6)  PTSD-SUD 39.73 (10.77) | Total 53.9% female, 46.1% male  PTSD 67% female, 33% male  SUD 39.7% female, 60.3% male  PTSD-SUD 47.6% female, 52.4% male | Not reported | Various | Alcohol and Drugs |
| Weiss (2022a), USA, Micro-longitudinal | Community adults who had experienced intimate partner violence in past 6 months | NA | | 145 | | 40.56 (11.60) | 100% women | Black or African American 31.3%, White 40.3%, American Indian/Alaska Native 8.3%, Hispanic or Latina 11.8%, Multiracial 5.6%, Not listed 2.1%, Prefer not to respond 0.7% | Intimate Partner Violence | Alcohol and Drugs |
| Weiss (2018), USA, Cross-sectional | Community - experienced intimate partner violence | NA | | 210 | | 36.14 (11.69) | 100% women | African American 48.6%, White 29.5% Latina, 16.2%, Another or multiple/racial ethnic background 5.7% | Intimate Partner Violence | Alcohol and Drugs |
| Weiss (2020), USA, Cross-sectional | Military veterans (Amazon M-Turk) | NA | | 468 | | 37.4 (11.41) | 70.5% male, 29.1% female, 0.4% female to male transgender | Ethnicity: Hispanic or Latino/a 25.2%, Not Hispanic or Latino/a (74.8%  Race: White 69.0%, Black 23.3%, Asian 6.0%, American Indian/Alaska Native 3.2%, Native Hawaiian/Other Pacific Islander 1.3%, Not Listed 0.9% | Not reported | Alcohol |
| Weiss (2021a), USA, Cross-sectional | Community - trauma exposed adults (Amazon M-Turk) | NA | | 320 | | 35.78 (10.63) | 50.3% men, 46.9% women, 2.8% different gender | Ethnicity: Hispanic/Latinx 14.3%, Not Hispanic/Latinx 85.7%  Race: White 81.6%, African American/Black 12.8%, American Indian/Alaskan Native 4.4%. Asian 2.8%, Different Race 2.6% | Various | Alcohol |
| Weiss (2019), USA, Cross-sectional | Community - trauma exposed adults (Amazon M-Turk) | NA | | 463 | | 35.66 (11.11) | 42.8% male, 55.7% female | Caucasian/White 76.6%, African American/Black 9.2%, Asian 11.2%, American Indian/Alaskan Native 4.5%, Native Hawaiian/Other Pacific Islander 0.6%, Hispanic 12.9% | Various | Alcohol and Drugs |
| Weiss (2013a), USA, Cross-sectional | Clinical – SUD inpatients | SUD-PTSD   SUD only | | Total: 205  SUD-PTSD:58  SUD only: 147 | | SUD-PTSD 35.72 (10.66)  SUD only 35.41 (10.27) | SUD-PTSD 71% male  SUD only 41% male | SUD-PTSD White 57%  SUD only White 55% | Not reported | Alcohol and drugs |
| Weiss (2013b), USA, Cross-sectional | Clinical - SUD inpatients | SUD-PTSD   SUD only | | Total: 93  SUD-PTSD: 38  SUD only: 55 | | 40.62 (9.68) | 76.3% male | Black/African American 60.2%, White 37.6%, Hispanic/Latino 2.2% | Childhood & Adolescent trauma | SUD patients - substance(s) not specified |
| Witte (2020), USA, Cross-sectional | College students | NA | | 946 | | 18.84 (1.06) | 71.9% women, 26.2% men, 1.9% missing | Not collected | Various | Alcohol |
| Wolitzky-Taylor (2023), USA, Cross-sectional | Adults with probable PTSD who reported hazardous drinking | N/A | | 513 | | 38.25 (10.07) | 49.9% female | Ethnicity Not Hispanic/Latino 85.6%, Hispanic/Latino 14.4%  Race White 76.8%, Black/African American 16.4%, Asian 2.7%, Native American/ Alaska Native 2.1%, Other 1.4% Native Hawaiian or Other Pacific Islander 0.6% | Various | Alcohol |

*Displayed as reported in included study.
